# Interdisciplinary approach to identify language markers for post-traumatic stress disorder using machine learning and deep learning

**DOI:** 10.1038/s41598-024-61557-7

**Published:** 2024-05-30

**Authors:** Robin Quillivic, Frédérique Gayraud, Yann Auxéméry, Laurent Vanni, Denis Peschanski, Francis Eustache, Jacques Dayan, Salma Mesmoudi

**Affiliations:** 1 PSL-EPHE, Paris, France; 2 ISCPIF, Institut des Systèmes Complexes, Paris île-de-France, France; 3 Laboratoire dynamique du langage, UMR 5596, CNRS, université ´ Lyon-II, Lyon, France; 4 Centre Hospitalier de Jury-les-Metz, centre de réhabilitation pour adultes, Metz, France; 5 UMR 1319 Inspiire, INSERM, Université de Lorraine, 9 avenue de la forêt de Haye, Nancy, France; 6 CNRS, UMR 7320 : Bases, Corpus, Langage, Nice, France; 7 Université PARIS 1 Panthéon-Sorbonne, Paris, France; 8 CNRS, CESSP, UMR 8209, Paris, France; 9 INSERM, NIMH U1077, Caen, France; 10 UNICAEN, Caen, France; 11 CHU de Rennes, Rennes, France

**Keywords:** Diagnostic markers, Statistics, Post-traumatic stress disorder

## Abstract

Post-traumatic stress disorder (PTSD) lacks clear biomarkers in clinical practice. Language as a potential diagnostic biomarker for PTSD is investigated in this study. We analyze an original cohort of 148 individuals exposed to the November 13, 2015, terrorist attacks in Paris. The interviews, conducted 5–11 months after the event, include individuals from similar socioeconomic backgrounds exposed to the same incident, responding to identical questions and using uniform PTSD measures. Using this dataset to collect nuanced insights that might be clinically relevant, we propose a three-step interdisciplinary methodology that integrates expertise from psychiatry, linguistics, and the Natural Language Processing (NLP) community to examine the relationship between language and PTSD. The first step assesses a clinical psychiatrist's ability to diagnose PTSD using interview transcription alone. The second step uses statistical analysis and machine learning models to create language features based on psycholinguistic hypotheses and evaluate their predictive strength. The third step is the application of a hypothesis-free deep learning approach to the classification of PTSD in our cohort. Results show that the clinical psychiatrist achieved a diagnosis of PTSD with an AUC of 0.72. This is comparable to a gold standard questionnaire (Area Under Curve (AUC) ≈ 0.80). The machine learning model achieved a diagnostic AUC of 0.69. The deep learning approach achieved an AUC of 0.64. An examination of model error informs our discussion. Importantly, the study controls for confounding factors, establishes associations between language and DSM-5 subsymptoms, and integrates automated methods with qualitative analysis. This study provides a direct and methodologically robust description of the relationship between PTSD and language. Our work lays the groundwork for advancing early and accurate diagnosis and using linguistic markers to assess the effectiveness of pharmacological treatments and psychotherapies.

## Introduction

There is evidence to suggest that language may play a role in the development and maintenance of PTSD. Studies using qualitative methods have shown that individuals with PTSD often have difficulty expressing their thoughts and feelings about the traumatic event and may use language in ways that are different from people without the disorder ^1–5^. Moreover, many of the psychotherapies recommended involve language from the defusing and debriefing stages onwards^6^. For example, a diagnosis scale based only on language markers (the SPLIT), has recently been proposed^7^. However, to the best of our knowledge, no study evaluates the diagnosis power of language on PTSD.

PTSD is a frequent endemic mental health condition that might develop after a person experiences or witnesses a traumatic event (Criterion A of DSM-5). In this work, we based our analysis on the symptoms of PTSD as defined by the DSM-5 (American Psychiatric Association. 2013). These symptoms are: being exposed to a traumatic event (criterion A of DSM-5), reliving the traumatic event (criterion B), avoiding reminders of the event (criterion C), negative changes in cognition and mood (criterion D), changes in physical and emotional reactions (criterion E), and the creation of distress or functional impairment in a person’s life (criterion G).

Establishing a diagnosis of PTSD and its symptoms has always been challenging in clinical practice owing to the clinical characteristics of the disorder itself, in particular the difficulty in confiding this experience, which is symptomatic of the cognitive and behavioral avoidance strategies for anything that might remind the patient of the trauma. On the one hand, some criteria may also fluctuate over time, creating “partial” PTSD in the nosographic sense, but just as disturbing for the patient. This is the reason why researchers have proposed and studied^8,9^ Partial PTSD (P-PTSD): recent studies analyzing the data from the attacks in Paris on the night of the 13th November 2015 also include P-PTSD in their cohorts^10,11^. On the other hand, comorbidities such as anxiety, depression, addiction, and social maladjustment, are often better identified than post-traumatic symptoms, which perpetuate a poor overall prognosis. The medico-economic consequences of increased morbidity and mortality in untreated patients are major^12^. Although effective pharmacological and psychotherapeutic interventions exist, and considerable progress has been made over the past two decades in shedding light on the biological effects, particularly through functional brain imaging, the absence of clinically available biomarkers remains a challenge. Can language analysis improve post-traumatic disorder diagnosis in practice?

Recent research in Natural Language Processing (NLP) has demonstrated that NLP can provide indicators of psychopathology, particularly for Depression, PTSD, Suicide, and Psychosis^13–15^. These Machine Learning (ML) or Deep Learning (DL) models might identify risk characteristics using spoken or written communications resulting in low-cost and low-effort healthcare systems^14^. This trend is possibly confirmed by the fivefold increase in the number of publications on mental illness detection using machine learning or deep learning methods over the last 6 years^16^. In spite of this tendency, there are no unified datasets or gold-standard methods to compare publications. Authors use different diagnostic tools and data sources to build NLP models that often lack interpretability ^17^.

Social media posts from Twitter (X) and Reddit associated with self-declared diagnosis are often used^18–20^, which enables the use of large datasets with a huge number of individuals but lacks homogeneity. Moreover, studies often compare exposed cohorts with PTSD to non-exposed cohorts without PTSD. Therefore, it is difficult to know if the linguistic markers are related to exposure to psychic trauma or to the diagnosis of chronic PTSD itself. Clinical notes^21^ and non-structural interviews^22–24^ are also used and often associated with a more precise diagnosis using self-questionnaire PCL-5 based on the DSM-5 criterion^25^ or semi-structured interview SCID (American Psychiatric Association 2013). To build NLP models, many kinds of linguistic features are extracted: statistical (number of words, number of words per sentence), morpho-syntactic (proportion of first-person pronoun, verb tense), topic modeling (LDA, LSA); word vector representation (Word2Vec, Doc2Vec, Glove, Fasttext), contextual embeddings vectors (BERT, Roberta), graph-based features^26^, coherence^27^ and readability features^28^, external resources such as LIWC^29^, sentiment analysis scores like LabMT^30^, TexBlob (Loria, 2018) or FLAIR^31^ and transfer learning methods like DLATK^32^ that used pre-trained models on social media data. The models used for the classification task, which consists of separating in people with and without PTSD, are mainly Random Forest (RF) ^33^, Logistic Regression (LR), CNN, LSTM, and transformers^15,16^.

The NLP and qualitative analysis seem to converge on a few language markers that characterize PTSD: (1) an overuse of first-person singular pronouns (I, “je”), (2) an underuse of third-person generic pronouns (it, “on”), (3) a greater number of words related to depression, anxiety, and death, (4) an overuse of spatial and temporal cues, (5) more negative emotions^19,34^.

**Figure 1:**
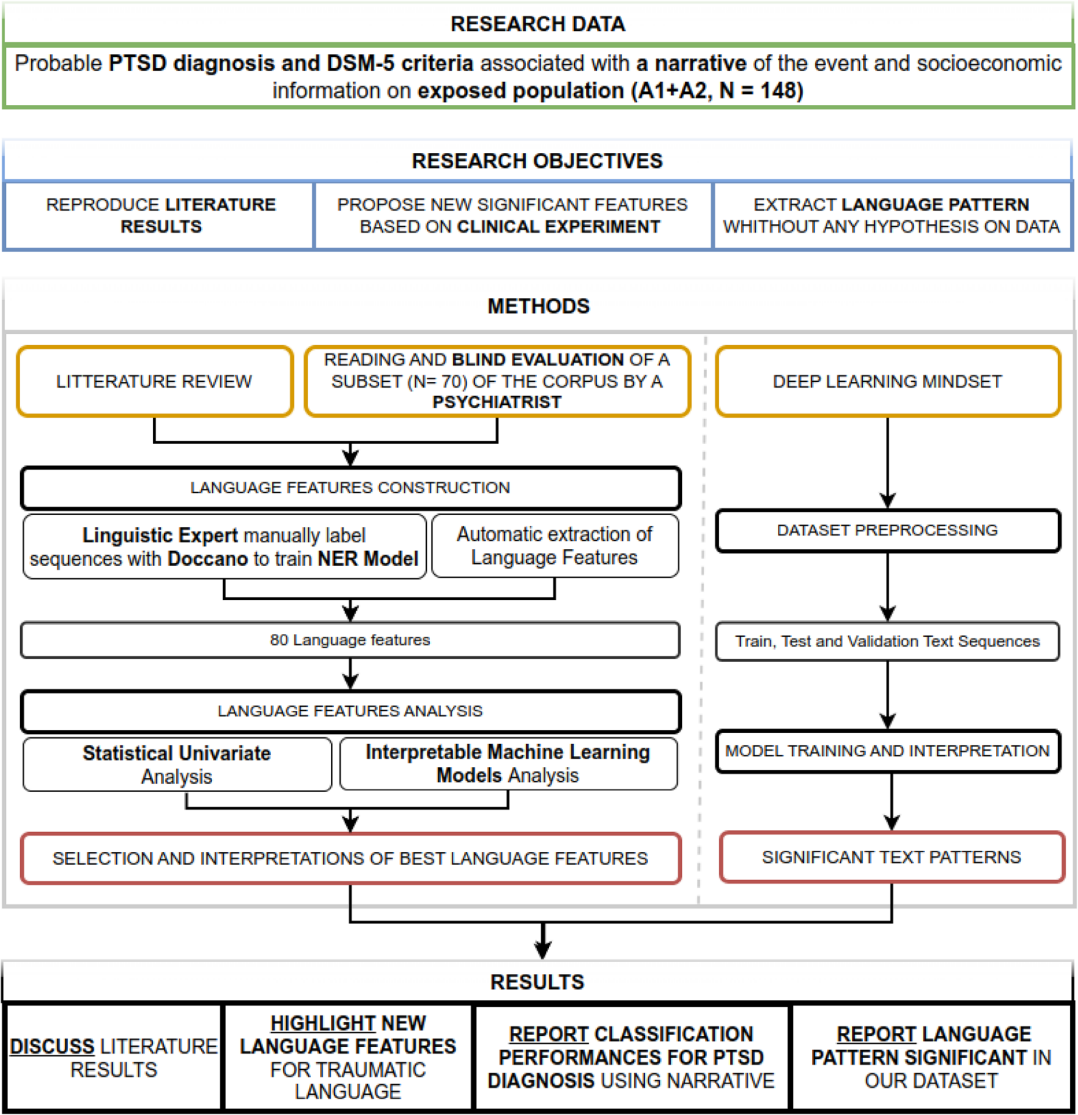
Graphical abstract that summarizes our methodological approach and contributions (A1: Direct Exposure, A2: Witnessing Trauma).

**Figure 2:**
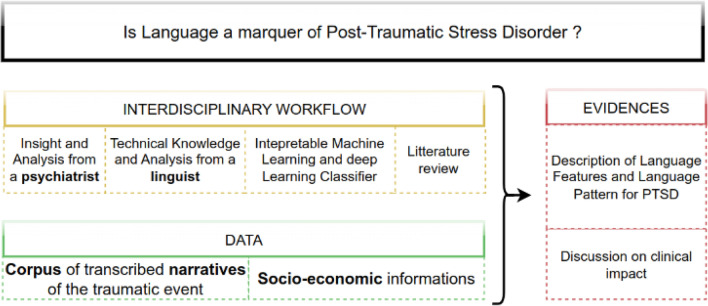
Description of the research mindset adopted in this article. The evidence presented is the result of an interdisciplinary workflow applied to an original dataset.

However, although the results seem compelling, these techniques have not been translated into clinical decision support systems^35^ because of their lack of interpretability, transparency, and generalizability^36^ (the degree to which the results can be applied to a broader context). This study is a first step towards tackling these challenges by presenting an explainable and transparent NLP-based PTSD assessment algorithm from incorporating language features. Our contributions are the following:

1. We report the first data on how well a psychiatrist can infer PTSD and symptoms solely from reading transcriptions.
2. We propose an interdisciplinary workflow (see Figs. 1 and 2) using different modeling mindsets: Statistical Frequentist Approach to describe associations, Explainable Machine Learning to decrypt the interaction among the features and measure the inference capabilities, and Interpretable Deep Learning to explore new language patterns.
3. We describe the link between language and PTSD using an original dataset that enables us to describe all the PTSD’s DSM-5 criteria in a homogenous population.
4. We have designed new methods and adapted existing ones for language feature extraction for French corpora and implemented them in Python. The implementation is accessible and re-usable on another dataset here (see Supplementary Table S0 in Supplementary Material).

## Methods

### Data

On the 13th of November 2015, three coordinated terrorist attacks occurred in Paris and its suburbs. The targets were the Bataclan concert hall, nearby trendy cafes in the 10th and 11th districts of Paris, and the *Stade de France* in a close suburb of Paris. It caused the death of 130 people, among which 90 were in the Bataclan, and it physically wounded 413 individuals. The data used in this study were collected by the “Etude 1000” protocol which includes collecting the narratives of 934 volunteers who were at varying distances from the attacks (from survivors to ordinary citizens of a provincial town). The particular and innovative methodology is based on three questionnaires submitted to the volunteers (cf Supplementary Fig. S1 in SM), most of which were videotaped. Moreover, this longitudinal study will take place four times, in 2016, 2018, 2021, and 2026. The present analysis focuses on the data collected in 2016^37,38^. In our research, we particularly focused on untrained exposed people, thus, we selected only the participants that verified the DSM-5 criterion A1 (directly experiencing the traumatic event) or A2 (witnessing or having been threatened at the time of the attacks). Table 1 provides a socio-demographic description of this particular sub-sample (N = 148).

**Table 1.** Socio-economic information of our cohort and statistical association with criterion A. In our cohort, criterion A is reduced to (A1: direct exposure, A2: witnessing the trauma)

Variable	A1	A2	p value	SE	Power
N	110	38	–	–	–
% Female	55.5	68.4	0.22	0.10	0.22
Age (year), mean ± std	37 ± 9	42 ± 12	0.01**	0.04	0.93
Students (%)	3.6	7.9	0.03*	0.3	0.86
Retired (%)	0.9	0			
Unemployed (%)	7.3	2.6			
Employee (%)	8.2	2.6			
Intermediate Profession (%)	20.9	15.8			
Executive and higher profession (%)	48.2	52.6			
Worker (%)	0.9	0			
Craftsman and merchant (%)	3.6	18.4			
Master degree or higher	59.1	57.9	0.42	0.14	0.28
Bachelor degree or equivalent	27.3	21.1			
High School diploma or less	11.8	21.1			
Single (%)	61.8	63.2	0.96	0.005	0.05
Living alone (%)	67.3	68.4	0.93	0.003	0.05

Both active and passive recruitment methods were used (see “Recruitments and Training” section in SM). Participants were recruited voluntarily, through information given by newspapers, the city hall of Paris, and associations of victims. Active recruitment involved contacting potentially eligible individuals by the program team. Participants were included from April 1st, 2016 to November 10th, 2016^1^, 5–11 months after the traumatic event. Filmed interviews took place in the studios of either the French National Audiovisual Institute (INA) or the Defense Communication and Audiovisual Production Agency (ECPAD). The film interview, divided into two parts, collects data about emotional, physical, and social reactions. In the first part of the interview, the participants were asked to produce a narrative relating to their experience of the attack (Q1) and also produced analysis on causes and consequences (Q2). It was followed by a shorter structured interview, an emotional memory questionnaire, that included 16 closed and some open-ended questions about post-traumatic symptoms and behaviors according to DSM-5^39^. In this study**,** we manually selected the answer to the first question of the semi-structured interview: “To begin with, could you tell me about November 13, 2015?”, we truncated the narrative of the traumatic event after the person had returned home or was taken to the hospital. We provided how informed consent was collected in the supplementary information.

PTSD diagnosis was assessed by professional psychiatrists specialized in PTSD using the answers from a semi-standardized trauma interview that enabled us to retrieve probable DSM-5 criterion, provided in SM of this study. The semi-standardized trauma interview was built by professional psychiatrists and its metrological qualities were assessed by comparing it with the results of the Structured Clinical Interview for DSM-5 (SCID) from a subsample (N = 85/934, see Supplementary Table S4). Participants were diagnosed with PTSD in its partial form (n = 42) if they had re-experiencing symptoms (criterion B), that caused significant distress and functional impairment (criterion G).

The interviews were transcribed using the Voccapia (Speech to Text API | Vocapia) software and then corrected by a human annotator. The corpus we examine is made up of 148 interviews and counts 849 725 words. On average, an interview is 5553 words long (∓ 3628 words).

### Ethical statement

Informed consent was obtained from all subjects, details on the procedure is presented in SM. The “Etude 1000”, component of the Program, was agreed upon by the “Comité d’évaluation éthique Inserm-CNRS (IRB) n°16321 which approved all experimental protocols. All methods were carried out in accordance with the EU general data protection regulation (GDPR).

### Informed consent statement

Before they participated in this study, all participants were provided with a detailed explanation of the research objectives, procedures, potential risks, benefits, and their rights as participants. All participants provided their voluntary informed consent by signing a consent form indicating their understanding of the study and their willingness to participate. It was explicitly communicated that participation was entirely voluntary, and participants were assured that they could withdraw from the study at any time without facing any consequences. Their confidentiality and privacy rights were emphasized, and they were made aware of how their data would be collected, stored, and used. The informed consent form is available upon request from the corresponding author.

### Language features (LF)

From Literature and expert knowledge (see Table 11), we formulated assumptions concerning the aspects of language that might characterize PTSD and associated symptoms. We then translated these hypotheses into quantitative measures that we extracted using human annotation and common NLP resources. We call these measures Language Features (LF) and regroup them in Table 10 according to the language aspect they concern. For instance, the proportion of present tense verbs in a narrative is a morphosyntactic Language Feature. A detailed and exhaustive list of all LF is available in the supplementary information, Supplementary Table S1 with their respective implementations.

- Sentiments and Emotion Features (n = 27), there is a rich literature exploring the link between emotion-related words or scores and PTSD narratives. Negative emotions are often associated with PTSD^17,18, 20, 40^. We used several external resources to compute scores associated with emotions, the French LIWC ^41^, composed of 64 categories, from which we selected only 4 categories (liwc_death, liwc_body, liwc_positive_emotion, liwc_negative_emotion), the EMA database^42^, is composed of 10 categories, the FEEL lexicon^43^ composed of 6 categories (fear, anger, surprise, sadness, disgust, and joy), the EMPATH lexicon^44^ composed of 36 semantical and emotional categories and finally, the LabMT dictionary^30^ translated into French that constructs a score of happiness. We also computed the Textblob^45^ polarity and subjectivity scores (Tutorial: Quickstart—TextBlob 0.16.0 documentation).
- Lexical Features, (n = 4) were extracted using a fine-tuned token classifier based on the CamemBERT^46^ architecture using the spacy-transformer template from spaCy. We constructed our dataset by manually labeling the documents using Doccano (https://github.com/doccano/doccano), an open-source tool for collaborative human annotation. The annotations were made by an experienced linguist. The models were trained using 200-word length sequences, 70% of the data were used for the training, 20% to test and fine-tune hyperparameters, and 10% to evaluate the performances of the model. In order to ensure correct performance evaluation, the evaluation sequences were taken from documents that were not used during the training. In Supplementary Table S3, we describe the dataset used and the performances for each task.
  - Lexical fields of death, body, physical sensations, and perceptions: using LIWC^29^, a previous study found that a larger number of sensory/perceptual words in trauma narratives was associated with PTSD^22,47^. However, as pointed out by the authors, LIWC has several limitations and might be easily wronged by idiomatic phrases. In our dataset, in the sentence: “Ils sont venus de derrière”, (“They came from behind”), the word “derrière” (behind) was associated by LIWC to the body but should have been associated with spatial words. In the same process as before we trained custom NER models for these categories: death, body, verbs of sensory perception, words of sensory perception, and physical sensation (see Supplementary Table S3).
- Morphosyntactic Features (n = 18), the proportion of pronouns and verb tenses were extracted using the stanza python library^48^, and customs NER models were trained using the same procedure as described in lexical features, in order to extract the values of present tense and third personal pronoun. See Supplementary Table S3, for performances and training data information.
  - The proportion of Pronouns and verb Tenses, which proposed a model trained on the French-gsd (GitHub—UniversalDependencies/UD_French-GSD) dataset that enables us to retrieve the Part-Of-Speech and Morphological characteristics. Therefore we computed the proportion of present, past, future, and conditional tenses for each narrative, as well as the proportion of first (singular and plural), second, and third personal pronouns.
  - Values of the present tense: Based on the literature review^49^, several studies^40,50, 51^ observed an increased use of the present tense among the narrative of PTSD groups. The morpho-syntactic features will enable us to confirm this observation on our dataset. However, the present tense might have different meanings depending on the context. It can have a historical value, referring to the past, and it also makes the speech more alive. Another meaning is generic, to express general truths like definitions or properties. Finally, it can have an enunciation value by referring to the present moment, to describe an ongoing action. These different values of the present tense can only be differentiated by the context. This is the reason why models based on contextual embedding should be relevant to differentiate them ^52^.
  - Values of “on” pronoun: As present tense, some pronouns can have different meanings according to their context; the generic pronoun plays an important role in trauma narratives ^1^. In our study, we investigate the different values of the “on” pronoun. It can be used as “we”, for example: “On est entré au Bataclan à 20h45” ("We entered the Bataclan at 8:45 pm"). But it can also be used as a synonym for ‘someone’: “On m’a marché dessus” (“Someone stepped on me"). Finally, it can be used generically: “on n’est jamais mieux servi que par que par soi même” ("you are never better served than by yourself".)
- Syntactic Features:
  - Passive Voice Features (n = 4), in the literature, the use of passive voice was associated with Mild Depression ^53^ but its association with PTSD has not been described so far. Yet, the grammatical passive voice is a marker of agency which is widely studied in the trauma literature ^54^. No tools were available to assess the passive voice in a French text, so we developed our tool, using an English tool^55^ as a model. Based on the linguistic literature in spoken French, we developed a rules-based algorithm and manually annotated 2530 passive voices from 25 narratives of our dataset. The performances are reported in Supplementary Table S2.
- Speech Disfluency Features (n = 9), the literature analyzing the phonetic data, shows that language disfluencies (fillers, repetition, hesitations, repairs, false starts, prolongation, etc.) are associated with psychosis ^56^ and PTSD ^57^. In this study we cannot work on speech data; however, it was possible to retrieve some disfluencies from the transcription such as the repetition of syllables, the false starts, hesitation, and silent breaks marked by “…” after a word or between 2 words (score_disfluencies), and the fillers by using a list of the most used in French (“euh”, ah, bah, etc.). All the features were normalized by the length of each testimony.
- Readability Features (n = 5), measures assessing the linguistic complexity or the readability of spoken or written productions have been widely applied to mental health detection^58,59^ but rarely to PTSD characterization. In our study, we use the readability toolbox^60^, see Supplementary Table S1 for the details of features.
- Graph Structure Narrative Features (n = 12), were extracted following the methods presented in Mota^67^, which represented textual data in graphs and using graph-theoretical tools that might be able to capture specific features of the flow of thought. As presented in supplementary materials, connectivity measures, transitivity, and the number of loops in 1,2,3 nodes, etc. were computed for each narrative.

### Analysis

#### Psychiatrist’s blind analysis

An initial assessment of the study involved a psychiatrist with no prior knowledge of the participants. From the entire dataset, we carefully handpicked 70 cases that are representative in terms of exposure and diagnosis, which accounted for approximately 50% of the global dataset. The clinician analyzed the narratives of the traumatic events, evaluating each criterion and making an independent diagnosis. In instances where no conclusive evidence was found, we opted to consider it as absent. The purpose of this section of the study is not to evaluate the psychiatrist's effectiveness in diagnosing PTSD, but rather to examine the amount of information presented in a transcription that can be used to identify PTSD symptoms.

Statistical analysis, was conducted to describe demographic, clinical, and text features between two (with and without probable PTSD; with and without each symptom taken separately) or three groups (probable PTSD, probable partial PTSD, no PTSD). In the first case, we used a Mann–Whitney U test^61^, and in the second, we used a one-way analysis of variance (ANOVA). We followed the literature’s recommendations^62^ concerning the Statistical Power (P) and Effect Size (ES). We used a chi2-contingency statistical test for categorical features (sexe, profession etc.)

Machine Learning (ML) analysis, was conducted to complete the previous statistical analysis that cannot capture complex relations between our features.

1. Model selection: To provide a comprehensive analysis, we chose three different models interpretable by design, thus enabling us to retrieve the critical features. The hyperparameters of the models were selected using grid search algorithm (more information in the experimental report presented in the SI).
  Logistic Regression (LR), is a commonly used model for classification problems due to its simplicity and model interpretability. To avoid overfitting, we used elasticnet regularization (l1_ratio = 0.6 and C = 0.1).
  Random Forest (RF), which is an ensemble algorithm based on decision trees^33^. Using low-correlated weak models can produce ensemble predictions with high accuracy. We choose parameters to avoid overfitting on a small dataset. (n_estimators = 40, min_sample_split = 0.4, min samples_leaf = 15)
  Explainable Boosting Machine (EBM^63^), is a glass box model, designed to have accuracy comparable to state-of-the-art machine learning methods like Random Forest and Boosted Trees while being highly intelligible and explainable. EBM is a generalized additive model (GAM) with few major improvements. First, EBM learns each feature function, using modern machine-learning techniques such as bagging and gradient boosting. The boosting procedure is carefully restricted to training on one feature at a time in a round-robin fashion using a very low learning rate so that feature order does not matter. Second, EBM can automatically detect and include pairwise interaction terms. The parameters were the following: max_leaves = 10, min_samples_leaf = 15, max_bins = 20 and early_stopping_rounds = 20.
2. Features selections. To avoid overfitting and training difficulty we reduced the number of features. Among the sentiment and emotional features, many were colinear. Hence, we only kept the sentiment features based on the emotional valence, which enabled us to keep only 6 sentiment features (Textblob, feel_positive, labMT, and liwc_emo*). The features concerning the passive voices were redundant, we only kept the normalized count of the passive voice. With these 2 rules, we kept for ML analysis only 55 features out of the 80 built. In SI, we evaluate the impact of the removed features on classification performance (Supplementary Fig. E1).
3. Training procedure. We split our data set into training and testing datasets (train: 80%, test: 20%), using stratified sampling on the exposition (criterion A from DSM-5) and probable diagnosis. We used the training dataset to select the best training configuration. We augmented the training size using the synthetic minority oversampling technique (SMOTE^64^) because we have an under-represented label (label: no PTSD). Then we computed the receiver operating characteristic (ROC) scores on the test dataset for 3 interpretable ML classifiers. The final scores were averaged over 100 separate randomized runs to demonstrate the robustness and stability of the results.
4. Interpretation. The models chosen were easily interpretable, we selected the models that achieved the best average performances over 100 random runs and we averaged the feature importances over these 100 runs.
5. Error analysis. On the same principle, we identified the 20 documents that are, on average, the most misclassified by the best models. We conducted statistical analysis on this subgroup to identify their specificity and enhance our model interpretations.

#### Deep learning analysis

To complete our analysis, we propose a sub-study based exclusively on a deep learning mindset that enables us to extract language patterns without apriori on our dataset. Indeed, the feature engineering described previously is particularly effective on small-size datasets but comes with many assumptions and hypotheses.

We employed a Convolutional Neural Network (CNN) model to solve our binary classification problem. To address the class imbalance in our dataset, we used a focal loss function^65^ with alpha = 0.7 and gamma = 2. The CNN model had an embedding size of 128, the vocabulary size was fixed to 2000, a hidden size of 32, and a kernel size of 9. To prevent overfitting, we employed several regularization techniques, including spatial dropout, L2 regularization, and early stopping. We trained the CNN classifier on a maximum of 50 epochs using annotated data from a training set (80%), and a validation set (20% of the training set) was used to monitor training. The performances were computed on a separate testing set (20%). The performance evaluation used the ROC-AUC scores averaged over 100 random runs.

To interpret the CNN classifier's predictions, we used two techniques: Class Activation Mapping (CAM) and Deconvolution. CAM highlights the important regions of the input that contribute to the classification decision^66^, while Deconvolution visualizes the contribution of each input feature to the final classification score ^67^. We followed the methodology proposed by ^68^ to apply these techniques to textual data. These techniques provide insight into how the CNN classifier makes its predictions and aids in interpreting the model's output. To emphasize the interpretability of the pattern extracted, we also computed and interpreted a multi-channel text CNN ^69^ that produces a pattern composed of a lemma, full form, and Part-Of-Speech tags.

**Figure 3:**
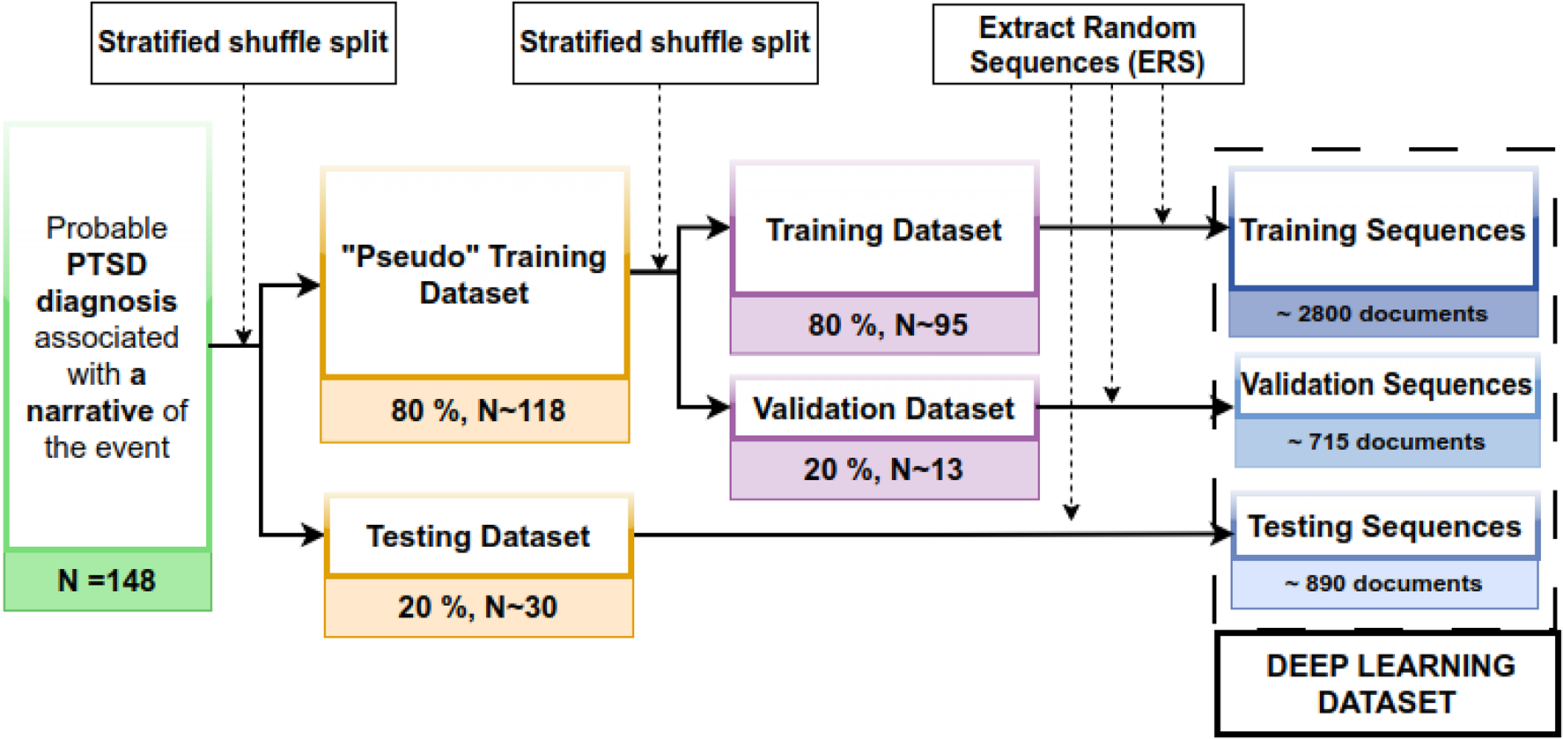
Description of the process of building the Deep Learning Dataset to avoid data leakage and ensure representative distribution.

**Figure 4:**
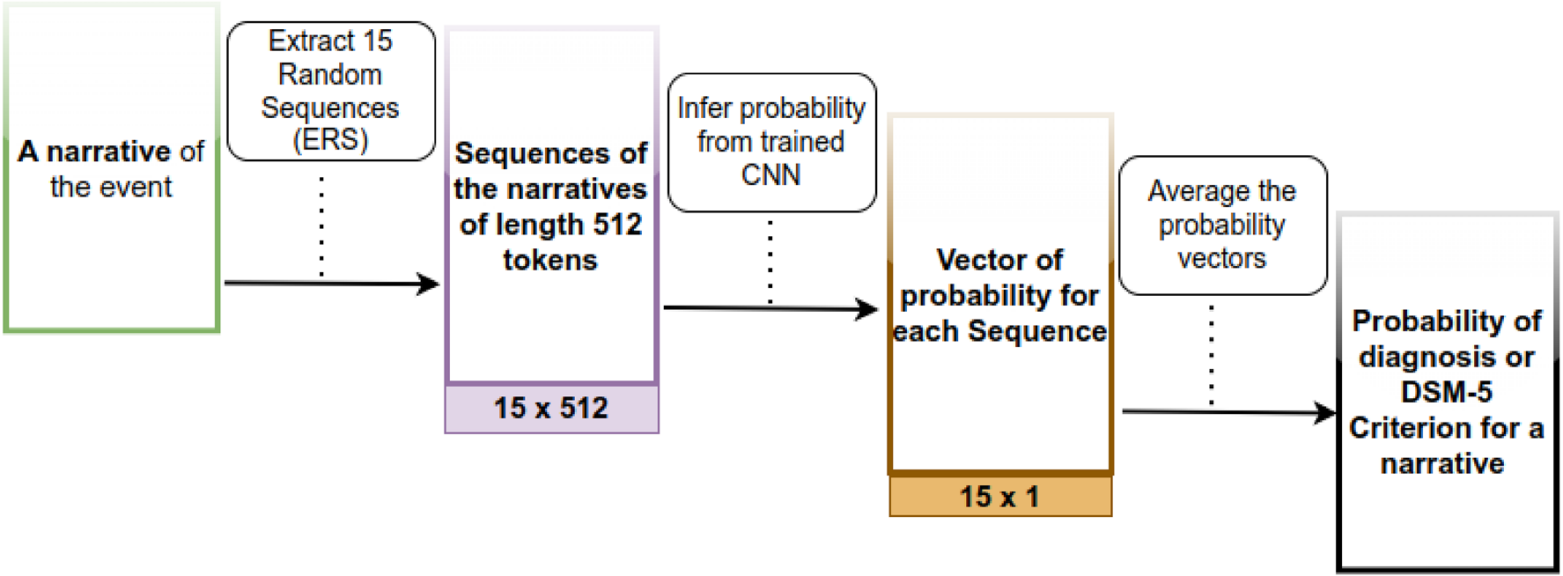
Description of the Inference process for a new document, using the CNN model.

The CNN models require the input to have the same length. We randomly extracted K (= 30) sequences of 512 tokens from each document. K was defined as the length of the 25% longest documents divided by the sequence length (512). The split into training and testing datasets was performed before the cutting of the sequence, avoiding data leaks. This process, as described in Fig. 3, is sensitive to random seeds and is slightly different over each training. The inference process described in Fig. 4 shows how we use the model to infer probable diagnosis on an unseen narrative by averaging the probability of all sequences.

Finally, the pattern extracted using the TDS scores will be qualitatively analyzed to propose future research directions.

## Results

Our objective was to examine the potential of language and narrative structure as indicators of Post-Traumatic Stress Disorder (PTSD). We conducted three complementary studies: Firstly, we employed a blind evaluation of a subset of the corpora by a psychiatrist, who specialized in psychotraumatology, which enabled us to evaluate how possible and easy it is to classify PTSD symptoms based on transcripts. Secondly, we sought to replicate the psychiatrist's evaluation through an extensive statistical analysis of text and narrative features, complemented by a highly interpretable machine learning approach, to characterize PTSD. Lastly, we introduced a hypothesis-free approach using a deep learning (text Convolutional Neural Network) classifier to identify potential language patterns that may have been missed by the previous methods. Supplementary Table S5 summarizes the results of the various approaches.

We will initially present the results of the univariate statistical analysis conducted on socio-demographic and psychopathological characteristics within our dataset. Subsequently, we will discuss the evaluation of the blind analysis made by a clinician. Moving forward, we will dive into the analysis of language features by presenting the key findings of the univariate statistical analysis (complete tables are provided as SM), followed by an examination of the performance and interpretation of the machine learning classifier. Finally, we will provide a comprehensive description of the text CNN classification method and its corresponding interpretations.

### Demographic, psychopathological, and linguistic characteristics

As demonstrated in Table 1, only age is associated with criterion A and PTSD diagnosis. Except for this feature, there is no statistical association between criterion A and socio-economic information, nor, see Table 2, with PTSD diagnosis. The length of the interview is slightly associated with the diagnostic (p-value = 0.05, EF = 0.03, power = 0.97) but strongly associated with the exposition type (p-value = 5.10–6, EF = 0.12, power = 0.99): People who were directly exposed to the traumatic event produced longer narratives (see Figure S4 and S5).

**Table 2:** Socio-economic information of our cohort and statistical association with PTSD.

Variable	PTSD	Partial PTSD	No PTSD	p value	SE	Power
N	70	42	36	–	–	–
% Female	56.5	71.4	51.4	0.11	0.17	0.46
Age (year), mean ± std	36 ± 9	37 ± 10	42 ± 10	0.01	0.05	0.99
Students (%)	7.2	4.8	0	0.07	0.29	0.6
Retired (%)	0	0	5.4			
Unemployed (%)	10.1	2.4	0			
Employee (%)	5.8	11.9	2.7			
Intermediate Profession (%)	18.8	14.3	27.0			
Executive and higher profession (%)	44.9	52.4	54.1			
Worker (%)	0	2.4	0			
Craftsman and retailer (%)	5.8	7.1	10.8			
Master degree or higher	62.3	50.0	62.2	0.58	0.13	0.17
Bachelor degree or equivalent	21.7	33	24.3			
High School diploma or less	13.0	16.7	13.5			
Single (%)	67.7	67.7	48.6	0.14	0.17	0.4
Living alone (%)	25.6	30.9	49.6	0.04*	0.2	0.61

### Psychiatric blind analysis

Our first approach to evaluate the link between Language and PTSD was made by assessing the ability of a psychiatrist to blindly evaluate the DSM-5 criterion and PTSD diagnosis based only on the transcription. Table 3, highlights that the global diagnosis is inferred with relatively high precision and recall, as it achieves 0.72 ROC-AUC scores. With the exception of criterion C, which is poorly identified, the other criteria are identified with high precision and recall.

**Table 3:** Evaluation of the blinded rating of a human expert (clinician psychiatrist).

	Partial or Full PTSD	Criterion B (intrusion sx)	Criterion C (avoidance sx)	Criterion D (negative changes in cognition and mood)	Criterion E (Hyperarousal sx)	Criterion G (Functional significance sx)
Precision	0.76	0.88	0.53	0.80	0.90	0.85
Recall	0.77	0.71	0.53	0.77	0.88	0.63
Specificity	0.65	0.72	0.59	0.65	0.71	0.78
AUC score	0.71	0.71	0.56	0.71	0.80	0.71

### Language features statistical analysis (see Supplementary Table S1 in SM)

- Full or Partial PTSD is significantly (Table 4) associated with more death-related (DEATH, liwc_death), body-related, and physical-sensations words (model_BODY, model PHYSICAL_SENSATIONS), less lexical diversity (token_ratio_score, noum_ratio_score), more repetitive discourse (L2, L3 degree_average, PE, L3, average_clustering), longer narratives (words_number, sentence_number), higher use of passive voice (passive_count_norm), more disfluencies (disfluencies_score), and finally fewer positive emotions in narratives (labMT happiness score, gobin_positive_score, etc.).
- Intrusions Symptoms (criterion B), are associated (see Table 5) with significantly more death, and physical sensations vocabulary and the number of disfluencies is positively associated with the reviviscence symptoms.
- Avoidance Symptoms (criterion C) are only significantly associated with more body words (p = 3.10–3, SE = − 0.4, power = 0.7) more physical sensations words (p = 0.04, SE = − 0.4, power = 0.7), a higher proportion of auxiliaries in the narratives (p = 0.01, SE = − 0.5, power = 0.7) and higher use of past participles (p = 3.10–3, SE = − 0.5, power = 0.8).
- Negative changes in cognition and mood (criterion D) are significantly associated (see Table 6) with more death words, both the LIWC category and our custom model, a higher proportion of “on” pronouns used with someone's meaning (impersonally/generically), a higher proportion of pronouns in the narrative, a higher use of the first personal pronoun plural (we).
- Hypervigilance Symptoms (criterion E) are associated (see Table 7) with higher use of the passive voice, higher use of death and body related words, a reduced lexical diversity (noum_token_ratio, adverb_ratio_score), less polarity (textblob_polarity), fewer positive emotions (labMT, gobin_joy, feel_positive) and fewer negative emotions (feel_sadness, empath_angry, empath_fear, gobin_sadness, feel_anger), more repetitions (PE, L2, degree_average, average_shrotest_path g0), lesser use of logical connectors (score_generical_connector_matches), more future tenses (verb_indicatif_future) and longer narratives (words_number, sentences_numbers).
- Functional significance symptoms (criterion G), are associated (see Table 8) with longer narratives, higher use of the passive voice, higher use of death, physical sensations, and body-related words, less lexical diversity (token_ratio_score), more repetitive narratives according to graph measures (PE, L2, degree average), more first personal pronouns (I, me), and less emotional polarity (labMT score, gobin_positive)

**Table 4:** The significant associations between Language Features and probable full or partial PTSD diagnosis, using Mann–Whitney U statistical test.

Textual features	p value	SE (cohen)	Power
Words_number	1.10–2	− 0.5	0.7
Sentence_number	2.10–2	− 0.4	0.6
Significant emotional features			
LabMT_score	1.10–2	0.6	0.9
Feel_positive_score	7.10–3	0.6	0.9
Liwc_positive_emotion_score	9.10–3	0.5	0.7
Gobin_positive_score	1.10–2	0.5	0.8
Gobin_joy_score	1.10–2	0.6	0.9
Significant lexical features			
Model_DEATH	2.10–4	− 0.7	0.9
Model_BODY	2.10–3	− 0.6	0.9
Model_PHYSICAL_SENSATIONS	9.10–4	− 0.6	0.9
Liwc_death	1.10–2	− 0.3	0.4
Significant morphosyntactic features			
Model_GENERIC_PRESENT	3.10–2	0.5	0.7
Significant syntactic features			
Passive_count_norm	4.10–2	− 0.4	0.6
Significant speech disfluencies features			
Disfluencies_score	5.10–2	− 0.4	0.67
Significant readability features			
Token_ratio_score	1.10–2	0.5	0.8
Noum_ratio_score	2.10–2	0.5	0.7
Significant graph discourse features			
Graph_L3	1.10–2	− 0.3	0.4
Graph_L2	1.10–2	− 0.4	0.5
Graph_PE	2.10–3	− 0.4	0.7
Graph_avarage_shortes_path	2.10–2	0.5	0.9
Graph_average_clustering	5.10–2	− 0.5	0.7
Graph_degree_average	1.10–2	− 0.5	0.7

**Table 5:** Significant (Mann–Whitney U statistical test) associations between Language Features and intrusion symptoms (Criterion B).

Textual features	p value	SE (cohen)	Power
Lexical significant features			
Model_DEATH	5.10–3	− 0.6	0.7
Model_PHYSICAL_SENSATIONS	5.10–2	− 0.5	0.5
Liwc_death	5.10–2	− 0.5	0.4
Speech disfluencies significant features			
Score_disfluencies (…)	5.10–2	− 0.5	0.5

**Table 6:** Significant associations between Language Features and criterion D symptoms (Mann–Whitney U statistical test).

Textual features	p value	SE (Cohen)	Power
Lexical significant features			
Model_DEATH	5.10–3	− 0.4	0.7
Liwc_death	5.10–3	− 0.4	0.6
Morphosyntactic significant features			
PRON	2.10–2	− 0.4	0.6
PROPN (Proper Name)	2.10–2	0.5	0.7
Model_ON_someone	3.10–3	− 0.5	0.8
First_personal_pronoun_plur	3.10–2	− 0.4	0.6

**Table 7:** Significant associations between Language Features and Criterion E symptoms (Mann–Whitney U statistical test).

Textual features	p value	SE (Cohen)	Power
Sentences_number	6.10–3	− 0.6	0.7
Words_number	2.10–3	− 0.6	0.8
Sentiment and emotional significant features			
LabMT	2.10–3	0.6	0.7
Feel_positive	9.10–4	0.7	0.9
Textblob_polarity	6.10–4	0.8	0.9
Lexical significant features			
Model_DEATH	8.10–4	− 0.7	0.8
Model_BODY	9.10–3	− 0.6	0.7
Model_PHYSICAL_SENSATIONS	1.10–3	− 0.6	0.7
Morphosyntactic significant features			
Verb_indicatif_future	3.10–3	− 0.6	0.8
Significant syntactic features			
Passive_count_norm	3.10–3	− 0.5	0.5
Speech disfluencies significant features			
Score_generical_connector_matches	7.10–3	0.7	0.8
Readability significant features			
Token_ratio_score	1.10–3	0.8	0.9
Noun_ratio_score	3.10–4	0.9	0.9
Adverb_ratio_score	4.10–3	0.6	0.7
Discourse coherence significant features			
Graph_average_shrotest_path_g0	3.10–3	0.7	0.9
Graph_Parrallel_Edge (PE)	2.10–3	− 0.7	0.8
Graph_L2	1.10–3	− 0.4	0.5
Graph_degree_average	1.10–3	− 0.75	0.9
Graph_degree_std	1.10–3	− 0.7	0.8

**Table 8:** Significant associations between Language Features and Functional significance (Criterion G) Symptoms (Mann–Whitney U statistical test).

Textual features	p value	SE (Cohen)	Power
Words_number	1.10–2	− 0.5	0.6
Sentiment and emotional significant features			
Polarimot_negative	3.10–3	0.6	0.8
Liwc_positive_emotion	7.10–3	0.6	0.8
LabMT	1.10–2	0.7	0.9
Gobin_positive	1.10–2	0.6	0.9
Lexical significant features			
Model_DEATH	3.10–5	− 0.8	0.9
Model_BODY	5.10–4	− 0.6	0.9
Model_PHYSICAL_SENSATIONS	2.10–4	− 0.7	0.9
Liwc_death	2.10–3	− 0.4	0.5
Morphosyntactic significant features			
First_personal_pronoun_sing	3.10–2	− 0.4	0.5
GENERICAL_PRESENT	1.10–2	0.65	0.9
Significant syntactic features			
Passive_count_norm	2.10–2	− 0.5	0.7
Readability significant features			
Token_ratio_score	3.10–2	0.5	0.8
Discourse coherence significant features			
Graph_PE	1.10–2	− 0.4	0.6
Graph_degree_average	4.10–2	− 0.4	0.6
Graph_L2	1.10–2	− 0.3	0.3

### Machine learning analysis

Model Performance best performances were mainly achieved using Logistic Regression with ElasticNet regularization. Performances average on 100 random runs on the test dataset for each classification task and each model is presented in Table 9. The global diagnosis (AUC = 0.69 ∓ 0.09), the criterion D (AUC = 0.67 ∓ 0.1), the criterion E (AUC = 0.75 ∓ 0.14) and the criterion G (AUC = 0.70 ∓ 0.1) achieved comparable performances to the human expert. Criterion B (intrusion symptoms) and C (avoidance symptoms) are the least learned criteria, with respectively 0.63 ∓ 0.14 and 0.55 ∓ 0.09 AUC scores.

**Table 9:** Performances average on 100 random runs on the test dataset for each classification task and each model.

Task	Models	Roc-Auc score
Full or partial PTSD	**LR**	**0.69 ∓ 0.09**
	RF	0.66 ∓ 0.10
	EBM	0.61 ∓ 0.08
Criterion B (Intrusions)	**LR**	**0.63 ∓ 0.14**
	RF	0.55 ∓ 0.16
	EBM	0.54 ∓ 0.14
Criterion C (avoidance)	LR	0.54 ∓ 0.09
	**RF**	**0.55 ∓ 0.09**
	EBM	0.50 ∓ 0.09
Criterion D (Dissociation)	**LR**	**0.67 ∓ 0.1**
	RF	0.57 ∓ 0.1
	EBM	0.56 ∓ 0.1
Criterion E (Hyperarousal)	**LR**	**0.75 ∓ 0.14**
	RF	0.73 ∓ 0.12
	EBM	0.72 ∓ 0.14
Criterion G (Functional significance)	LR	**0.70 ∓ 0.1**
	RF	0.67 ∓ 0.1
	EBM	0.67 ∓ 0.1

**Figure 5:**
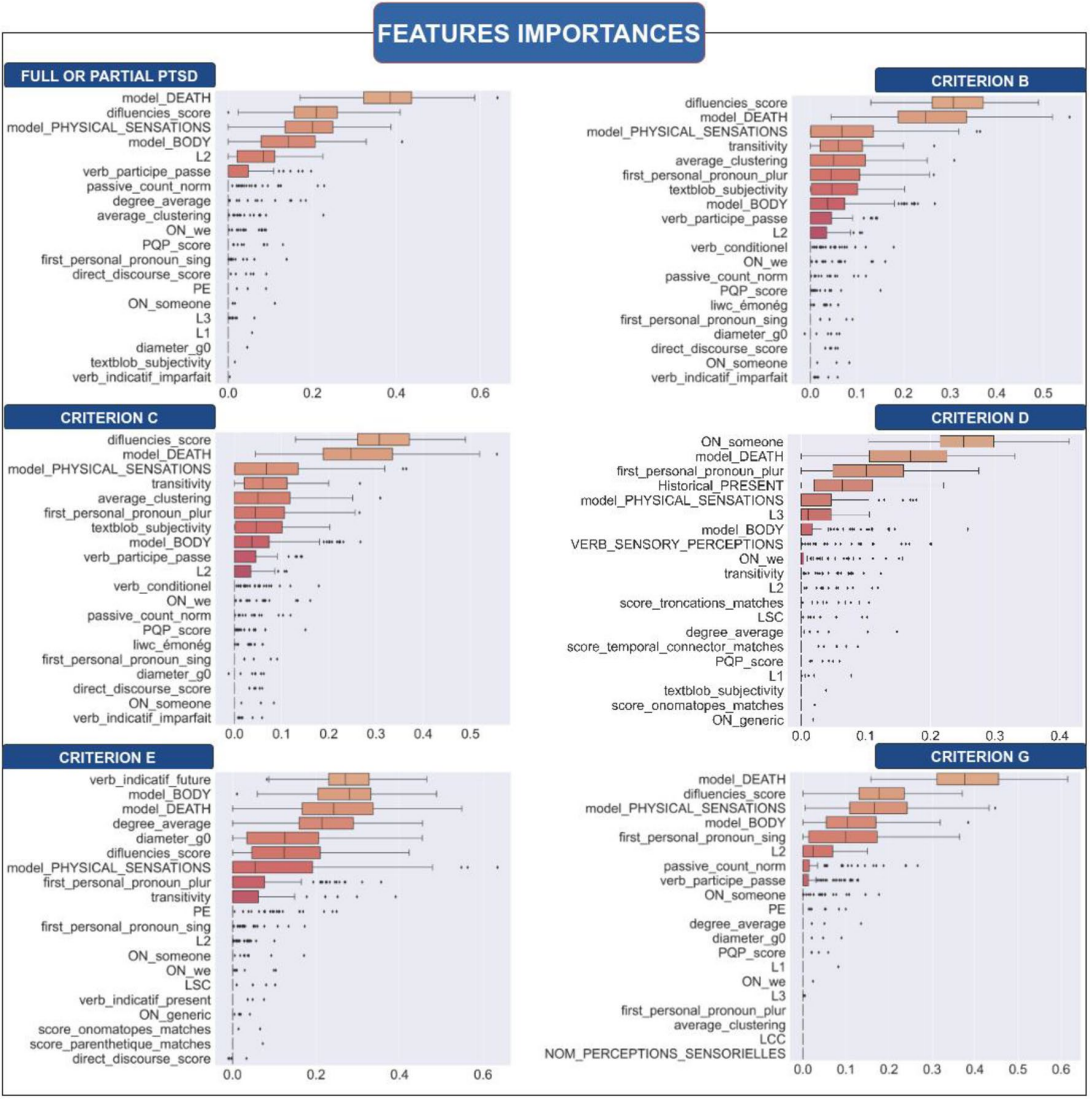
Features Importances of the diagnosis and all the criteria. The Feature importance is the average over 100 random runs for the model achieving the best results.


Model Interpretation, after selecting the best classifier for a classification task using the ROC-AUC score average over 100 random runs, we average the feature importance scores using the appropriate method for each kind of model, see Fig. 5. All the interpretations should be analyzed by taking into account the performances presented in Table 9 for each classifier. We found that the most important features, on average, for predicting:

- Probable full or partial PTSD, are the proportion of truncations in the text (“wor…”), the lexical field associated with death, physical sensation, and body, the verb conjugated to past participle, direct discourse, historical present, and L2 and average node degree for the graph features.

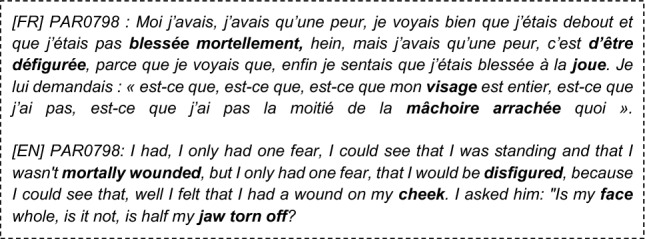

- Criterion B (intrusions), is, the proportion of disfluencies in the text (“wor…”), the lexical field associated with death, and physical sensation, the score of subjectivity from the textblob engine, and the transitivity and average clustering from the graph.

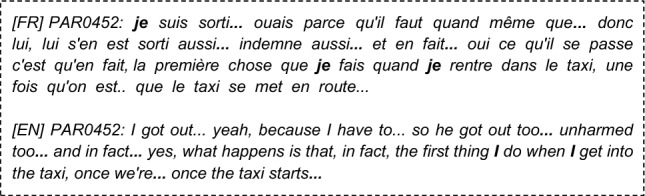

- Criterion C (avoidance), cannot be considered due to the performances’ classifier that is near random.
- Criterion D (negative changes in cognition and mood), are lexical fields associated with death and physical sensations, verbs of perception, first person plural pronoun, and “ON_someone”.

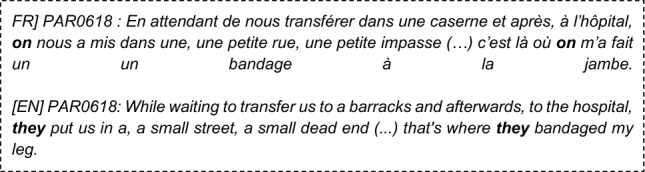

- Criterion E (hyperarousal), are lexical fields of death, body and physical sensation, the future tense, the verbs of perceptions, and the average degree of nodes.

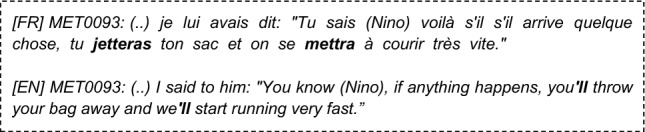

- Criterion G (functional significance), is the lexical field of death, body and physical sensation, the first person singular pronoun, and the proportion of truncations in the narrative transcription.

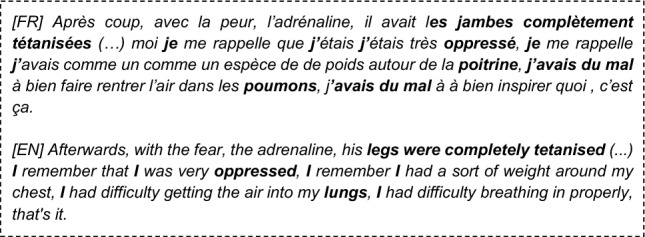


#### Error analysis

Statistical tests were carried out on the 20 most frequently misclassified documents over the 100 runs by the best classifier. The results show some statistical associations with the socio-demographic variables. The errors concerning the full or partial PTSD classifier are associated with the way of residence (p-value = 4.10–2, EF = 0.16, Power = 0.6). The classifier makes significantly fewer mistakes on people living alone. The errors for criterion B (intrusion symptoms) were associated with the type of exposition. We make significantly more mistakes in the A2 group (p-value = 4.10–5, EF = 0.3, Power = 0.98). The errors for criterion D (negative changes in cognition and mood) were associated with qualifications (p-value = 6.10–3, EF = 0.4, Power = 0.95) and matrimonial situation (p-value = 3.10–2, EF = 0.4, Power = 0.8). Indeed, the vast majority of mistakes concerned the “bac + 4 or more” subgroup and within the single population. The errors for criteria E (hyperarousal symptoms etc.) and G (functional significance etc.) were associated with the type of exposition. We make significantly more mistakes in the A2 group (p-value = 3.10–4, EF = 0.3, Power = 0.9) and respectively for the criterion G (functional significance etc.) (p-value = 3.10–3, EF = 0.2, Power = 0.9).

### Deep learning analysis

#### Models performances

The CNN classifier performs worse than the psychiatrist or the machine learning classifier (see Supplementary Figs. S7 and S8). Particularly criteria C (avoidance), and D (negative changes in cognition and mood) are almost not learned on average and the performances on criteria B (Intrusion), E (hyperarousal), and G (functional significance) are nearly 0.1 down compared to other approaches. These underperformances might be explained by several reasons. Firstly, when constructing the dataset, the sequence breakdown can create false positives if the traumatic discourse is not uniformly distributed throughout the discourse. Secondly, previous approaches were guided by clinical knowledge that allowed us to construct relevant features and might explain the over-performances (Table 10).

**Table 10:** Example of significant language pattern extracted by the CNN model.

Pattern in French	Pattern in English
*que … C' est un peu de ma faute … " Surtout que c' était ce était … En plus … j' ai oublié de parler*	*that … It's a bit my fault… "Especially as it was … Plus … I forgot to speak*
*était étendu par terre , donc … face contre terre et le visage … en sang , enfin je pouvais …*	*was lying on the floor, so… face down and… bleeding, well I could…*
*, mais , bon , ils s' occupaient des blessés et des morts , quoi , pas de*	*but, well, they were looking after the wounded and the dead, not the*
*… donc dans la fesse , et je me souviens , pareil , il réalise pas , je … je*	*… so in the buttock, and I remember, same thing, he doesn't realize, I… I*
*le sang en fait … d' Adrien … moi je pensais qu' il allait mourir , parce que*	*the blood in fact … of Adrien … I thought he was going to die, because*
*le coup euh … bah voilà Marie est décédée et puis … dans … c' était très fouillis , je me souviens*	*the blow … well, Marie died and then … in … it was very messy, I remember*
*se passer , pourtant si … euh … Donc euh … donc voilà on court*	*happen, although if … er … So um … so here we are running*
*disant : " Non non c' était … c' était rien , c' était … des noeuds ou*	*saying: "No, no, it was … it was nothing, it was … knots or*
*due le coup … et euh … et je me souviens le … lui dire : "*	*of the blow… and er… and I remember the… telling him: "*
*mon cerveau , il avait un peu , " clack " , il avait … il avait débranché *	*my brain, it had a bit, "clack", it had … it had disconnected*

#### Model interpretations

Due to the lower performances of specific classifiers for each criterion, we focus on these parts on the interpretation of the main classifier that concerns full or partial PTSD. We selected a classifier that performs as well as the average performance and using the methodology proposed by Vanni et al.^68–70^, we extract the most representative sequence for the positive class Full or Partial PTSD and we can extract the most significant pattern using the Text Deconvolution Sailliency applied on a multi-channel architecture^69^. The sequence of 512 tokens with the higher classification probability is presented here (see Supplementary Table S6 for French). While reading this sequence and the pattern in Table 11, we can observe that the model particularly focuses on disfluencies (euh, …), repetitions, disorganized narrative, and onomatopoeia (‘clack’)‘. It also highlights direct discourse and some first names such as Adrien or Marie. Finally, we also observe some lexical fields that we expected such as death (“lying on the floor,” “the dead”), and body parts (“buttock”, “blood” and “my brain”). We also note numerous figures of speech (metaphors: “it had disconnected.”) and many words related to memory and memory impairments (“I remember”,” it was very messy”).

Most representative sequence translated into English: […] a relationship with death, in those cases … where well I'd seen others of the dead … in the Bataclan … and er … of the good time OK … Marie died, and it was more in relation to her family, where I was thinking about her family, saying to myself: "That's too stupid, she's made it this far and … well, it's at the first-aid post where she died, even though it's the … voilà. "So it's … but I didn't … I didn't think at the time that it could be me … that, it was months later that I could think things like that, but … not at the time. Er… at the time er… well, Marie died and then … in … it was very messy, I remember that the paramedics … you could feel that there was a bit of panic, it's … I saw them getting agitated but coming back to the same place fifteen times and poor Marie we came at least three, four times, to see if she was OK and then she was dead each time so um … so PRON, I, I remember, also thinking to myself that, normally, we make colour codes for that, so that … to identify the, the, the, the deceased, the … the seriousness of the injured. So um … so there you have it. And um … and at the moment when I really began to find the time long, I … I remember meeting the eye … I perhaps stood up, a little … and I met the eyes of a girl we were evacuating, and I remember that we looked at each other and that it was really … a connivance, to think: "What the fuck are we getting out of here? "To see … yes, the look in our eyes as we couldn't talk to each other, because we were at the other end of the courtyard, from each other, but really from … and then happy to catch someone's eye, too. So this girl left and then I was finally evacuated, but I was … there weren't many people in the yard when I was evacuated … So […].

**Table 11:** Description of the link between research hypothesis and Language Features.

Features group	Example of associated hypothesis	Hypothesis’ origin	Extraction method
Sentiments and emotions	Traumatic language is associated with more negative emotions	Literature extracted	NLP literature resources
Lexical	Traumatic language is marked with some specific lexical fields such as death, body or physical sensations	Expert knowledge and literature extracted	Human annotation to train NER model
Morphosyntactic	Traumatic language is marked by a specific use of pronouns and verb tenses	Expert knowledge and literature extracted	Human annotation to train NER model and literature resources
Syntactic	Traumatic language is associated with an overuse of the passive voice	Expert knowledge	Literature resources
Speech disfluencies	The disfluencies are more frequent in traumatic language	Expert knowledge	Regular expressions
Readability	The retranscription of narratives from symptomatic people is less easy to read	Literature extracted	NLP literature resources
Narrative coherence	People with PTSD symptoms tends to produce less coherent narrative	Expert knowledge	Literature resources

## Discussion

The blind evaluation provided evidence supporting the notion that language could serve as a marker of PTSD (ROC-AUC = 0.72), while the ML method achieved comparable performance to the clinician's prediction (ROC-AUC = 0.69), thus confirming and quantifying the language features associated with PTSD. The last study, utilizing the CNN, exhibited lower performance (ROC-AUC = 0.64) but revealed language patterns that were not captured by the previous approaches. Regarding the underperformance of Random Forest and Explainable Boosting Machine, it aligns with existing literature. Typically, Random Forest and Explainable Boosting Machine outperform logistic regression (LR)^34^. However, in text classification benchmarks, LR tends to outperform other ML model^71,72^.It is noteworthy that although the ROC-AUC scores may not reach 1, they are comparable to the AUC values reported in the literature when comparing two Gold-Standard diagnostic methods for PTSD (PCL-5 and SCID). For example, Nedelcea et al.^73^, reported an average ROC-AUC of 0.76.

Supplementary Table S5 indicates that we have identified a range of markers representing a linguistic system associated with PTSD symptoms. Specifically, all examined dimensions of language are disrupted. These disruptions were related to specific PTSD symptoms. Language features, including lexical characteristics (e.g., lexicons related to death, body, and physical sensations), emotional aspects (e.g., the emotional valence of discourse), morphosyntactic patterns (e.g., use of certain verb tenses and pronouns), syntactic aspects (e.g., the proportion of passive voice), and fluency-related elements (e.g., incomplete statements and repetitions), collectively contribute to identifying full or partial PTSD. Furthermore, discourse structures, as evidenced by graph-based features, are also altered in individuals with PTSD but further studies need to be carried out to provide more interpretable and theory-driven features for discourse structure.

Analyzing the sub-symptoms of PTSD based on the DSM-5 criteria, we found that reviviscence (Criterion B) is predominantly marked by lexical features, disfluencies, and perception-related verbs, which may indicate a literal re-experiencing of the traumatic scene. Negative changes in mood (Criterion D) is specifically identified by verbs of perception and the use of generic pronouns like "generic on" and "we," reflecting the depersonalization process. Hypervigilance, as represented by Criterion E, manifests through the use of perception-related verbs, future tense, historical present, and discourse that carries emotional intensity. The pronounced prevalence of the future tense among individuals experiencing symptoms of PTSD suggests that their anticipation of forthcoming activities diverges from that of psychologically sound populations, potentially stemming from involuntary future projections^74^. Impairment of global functioning (Criterion G) is linked to the frequent use of the first-person pronoun "I." This finding adds to the body of literature highlighting the excessive use of the first-person pronoun, a phenomenon commonly observed in individuals with depression, anxiety, and PTSD, which, in turn, is strongly associated with substantial clinical distress. It is worth mentioning that the difficulty in describing avoidance (Criterion C) may be correlated with this symptom and could reflect a bias in our dataset, as individuals participating in scientific studies may exhibit avoidance strategies.

Lexical features play a particularly discriminative role in the overall diagnosis of PTSD and each individual symptom. The mention of lexical fields related to death corresponds to Criterion A1 of PTSD, while the inclusion of lexical fields related to the "body" and "physical sensations" encompasses elements specific to the studied trauma (e.g., physical injuries, physical proximity in enclosed spaces) as well as physical manifestations of stress, anxiety, and negative emotions. We hypothesize that these lexical markers serve as reliable estimators of peritraumatic factors commonly assessed using tools like the Peritraumatic Distress Inventory^75^. One of the major peritraumatic risk factors associated with PTSD is loss of agentivity, which seems to be well captured by the proportion of passive forms in the narratives of our study. In contrast to the existing literature, we found no over-use of negative emotions. In our case, all the testimonials (PTSD or not) relate to a personal traumatic event, which could lead to homogeneity in the use of negative emotion terms. This is a major difference from the collections of tweets or reddit texts, in which other subjects were addressed by non-PTSD people^19,34^.

Additionally, graph-based features^26^ effectively capture the structural characteristics of PTSD language. Indeed, measures such as L2, L3, and PE reflect narrative repetition and logorrhea, while average node degree, transitivity factor, and clustering coefficient reveal structural differences in narratives. In the forthcoming work, we will propose further exploration to better describe and understand how PTSD’s discourse structure differs from that of non-PTSD.

The exploratory deep learning-based approach presented in this study uncovers the need for additional inquiry in unraveling the intricacies of traumatic language. Indeed, our qualitative analysis of the patterns extracted using TDS scores raises some considerations. First, there's the aspect of the low AUC score associated with the model. Second, there's the lack of quantitative analysis of the extracted patterns. Nevertheless, they provide avenues for further exploration. Specifically, we posit that figures of speech wield significant influence, yet their extraction using quantitative methodologies is challenged by their reliance on external world knowledge. Moreover, in alignment with the suggestions and practices of prior research^75,76^, we believe that a multimodal model incorporating speech and video data has immense potential in yielding insights in spite of drawbacks associated with data privacy.

Our research represents a notable advance in developing efficient language markers for PTSD. These markers provide low-cost, easily anonymized solutions with the potential to transform crisis response prioritization and patient monitoring. Their use will enable rapid identification of high-priority cases, thereby improving crisis intervention. They may also facilitate a thorough assessment of treatment effectiveness by tracking language markers during treatment, supporting more personalized patient care pathways and enhancing the objectivity of clinical judgment.

Methodologically, our study offers several contributions. First, we implemented a design that leverages the expertise of different professionals (psychiatrists and linguists) to produce scientific results. Second, by constructing highly discriminative and interpretable features using custom models based on expert knowledge, we demonstrated the potential of this approach for other pathologies, such as depression or psychosis.

### Limitations

Despite the meticulous application of our scientific methodology, certain limitations in our study could not be entirely mitigated. Firstly, the size of our dataset remains relatively small, which may restrict the generalizability of our findings regarding the association between language and PTSD, although it is one of the largest corpora in the existing literature. Additionally, the recruitment process employed might have introduced some bias into our sample, as individuals who experienced more intense trauma are often less inclined to participate in psychological trauma studies. We believe that a specific scientific design should be built to study precisely this dimension of avoidance. Particularly, the avoidance patterns might not have been well captured by textual data and could be better captured with video and audio data. This can also explain why clinical experts had difficulty in assessing this symptom using only transcriptions. Moreover, the study design did not encompass precise control over comorbidities and did not measure the effect of treatment, which could potentially influence the observed language patterns such as depression or anxiety. Moreover, as it is a time-consuming task, the blind assessment was only carried out by one psychiatrist, which weakens the results. Lastly, the NLP methodology developed was focused on interpretation and transparency, and more recent methods such as Large Language Model or deeper neural model can improve classification performance (experiments are reported in the SI using Mistral-7B^76^ and other architectures, Supplementary Tables E3, E4, E5).

## Perspective

Although our study represents a valuable advancement in the development of efficient language markers for PTSD, future studies could integrate multimodal data to emphasize the analysis of disfluencies and avoidance patterns. While our models show promise for diagnosing PTSD in survivors of terrorist attacks, further validation in diverse trauma populations is necessary to assess their broader applicability and to propose reliable clinical use. The first step would be to test the generalizability of our model to the rest of the Etude 1000 cohort (indirectly exposed individuals), and then to apply it to cohorts with different sources of PTSD (such as car accidents, war zones, or sexual violence). The next studies will also investigate how the linguistic markers can be used as predictive factors by exploring the second and third phases of the longitudinal Etude 1000, which took place in 2018 and 2021.

### Supplementary Information

Supplementary Information.

## Data Availability

The participants of this study did not give written consent for their data to be shared publicly, due to the sensitive nature of the research, then, supporting data is not available. However, the anonymized and non-identifying text features are available and can be downloaded using the link in Supplementary Table S0 SM. The anonymized Text features dataset is available on this GitHub repository: text_features.csv. The Anonymized psycho-socio-economic dataset: socio_psycho_dataset.csv.
