# Supplementary material for: Interdisciplinary approach to identify language markers for post-traumatic stress disorder using machine learning and deep learning
Source: Sci Rep. 2024 May 30;14:12468. doi: 10.1038/s41598-024-61557-7 (PMC11139884; doi:10.1038/s41598-024-61557-7)
Supplement: Supplementary file 1 — Supplementary Information. [file 41598_2024_61557_MOESM1_ESM.docx]

Supplementary Information

[Data, Code, and Reproducibility 0](#_332r9zv01ip7)

[Table S0. Supplementary Information concerning data, code, and reproducibility 1](#_4f0jzh1ymv2l)

[Recruitments and Training 2](#_9q4mhna9gqrz)

[Supplementary Figures 3](#_uw8n0gr6eiod)

[Figure S1: Description of the process to build the Research Dataset 3](#_5uwse2fn9dhs)

[Figure S2. Processing for computing Graph Languages Features 4](#_sxkmtlhquj2c)

[Figure S3: Multi-channel Classifier from Vanni et al. 2023. 4](#_ios0l0hd7g8p)

[Figure S4. Distributions of the number of words based on exposition type and PTSD diagnosis 4](#_krzec7qolmwt)

[Figure S5: Distributions of the number of words based on gender and PTSD diagnosis 5](#_45o1qfjl3h55)

[Figure S6: Description of multiple evaluation metrics for ML Classifiers 5](#_6qnl718k1h2y)

[Figure S7. Average ROC curves for each text CNN Classifier related to each DSM-5 Criterion 6](#_au6e3zao64ao)

[Figure S8. Average ROC curves for each text CNN Classifier related to each DSM-5 Criterion using sequences-based evaluations 7](#_3v00smesfvb)

[Supplementary Tables 8](#_q4ovki1mahk9)

[Table S1. - Exhaustive list and description of language features 8](#_o9vqwpyx8dfo)

[Table S2: Reported performances of detecting passive voice in our corpora using the custom spacy matcher 10](#_32cqgbu17kj6)

[Table S3: Dataset Size and Performances for each custom camembert-based token classifier models 10](#_tpdhphtqthem)

[Table S4. Metrologic validation of the trauma interview on the 85 participants of the “Etude 1000” who were diagnosed using SCID. 10](#_nwd2lyklaa6)

[Table S5. Synthesis of the results produced by statistics and machine learning analysis 11](#_ycvdyb2u5tql)

[Table S6. Not Translated most significant sequence associated with Full or Partial PTSD 13](#_89m1lueanmgb)

[Interview Material (Translated) 14](#_59bhts2jd6qt)

[First Part, open questions about the event 14](#_bvaz0c6d4j7y)

[Second Part, Semi-standardized interview for PTSD diagnosis assessment 15](#_pbii89bs1nnn)

[Experimental Report 21](#_amsbn9kz8yr9)

[Machine Learning Experiments 21](#_6lrmnnin7c7l)

[Deep Learning Experiments 24](#_9mhuy4y5ryxh)

[LLM experiments 25](#_e0g412xwvpoz)

[Bibliography 29](#_6mvk62pkkkqk)

# Data, Code, and Reproducibility

### **Table S**0**.** Supplementary Information concerning data, code, and reproducibility

| *Description* | *Access Link* |
| --- | --- |
| **Data** | |
| Text features dataset | [text_features.csv](https://github.com/psycholinguistics2125/linguistic_code_of_ptsd/blob/main/data/corpus/text_features.csv) |
| Anonymized psycho-socio-economic dataset | [socio_psycho_dataset.csv](https://github.com/psycholinguistics2125/linguistic_code_of_ptsd/blob/main/data/corpus/socio_psycho_dataset.csv) |
| Complete list of TDS pattern | [hyperdeep_pattern](https://docs.google.com/spreadsheets/d/1zbPxycdDrFAT_H1iyo7kIRiEFix_YgXPNP9_sUiqWLE/edit?usp=sharing) |
| **Application and visualization** | |
| Web application for data visualization | <https://13-november-data-viz.streamlit.app/> |
| Web application for computing passive voice | <https://passive-fr.streamlit.app/> |
| **Models** | |
| Token classifier for the value of present tense | <https://huggingface.co/binbin83/fr_present_tense_value>  DOI: 10.57967/hf/1189 |
| Token classifier of the value for the pronoun “on” | <https://huggingface.co/binbin83/fr_on_value>  DOI: 10.57967/hf/1188 |
| Token classifier models for Death | <https://huggingface.co/binbin83/fr_lexical_death>  DOI: 10.57967/hf/1186 |
| Token classifier models for Sensation and body | <https://huggingface.co/binbin83/fr_sensations_and_body>  DOI: 10.57967/hf/1187 |
| **Code** | |
| Detection of passive voice | [NLP-for-Psycholinguistic/passive_detection_app](https://github.com/NLP-for-Psycholinguistic/passive_detection_app) |
| Extract features from text | [psycholinguistics2125/text_feature_toolbox](https://github.com/psycholinguistics2125/text_feature_toolbox) |
| Finetuning CamemBERT model with spacy | [psycholinguistics2125/train_NER](https://github.com/psycholinguistics2125/train_NER) |
| Reproduce ML and stats results | [psycholinguistics2125/linguistic_code_of_ptsd](https://github.com/psycholinguistics2125/linguistic_code_of_ptsd)  DOI: 10.5281/zenodo.8414555 |
| Reproduce DL results | Performances: [NLP-for-Psycholinguistic/deep_text_classifier](https://github.com/NLP-for-Psycholinguistic/deep_text_classifier)  Interpretation: [psycholinguistics2125/hyperdeep](https://github.com/psycholinguistics2125/hyperdeep) |

#

# Recruitments and Training

All the participants were volunteers and were recruited through information provided by newspapers, Paris City Halls, and victims' associations. To facilitate the recruitment of volunteers, a mediation team was established. Their role involves identifying potential volunteers, providing them with information about the program's objectives, ensuring that they have received all necessary information, inviting them to participate, scheduling meetings for recruitment, and maintaining communication with the volunteers. Volunteers can get in touch with the mediators through four different channels,

1. Face-to-face contact: Mediators introduce the program to potential volunteers during street flyer distributions and other field operations.
2. Email communication: Mediators receive and respond to messages sent to "participation@memoire13Novembre.fr," subsequently contacting the volunteers.
3. Phone calls: The mediation team manages two telephone lines, allowing volunteers to inquire and sign up for the program via phone.
4. Sign-up during public meetings: During public meetings, events, or association gatherings, the mediation team provides contact sheets that enable them to re-establish contact with the volunteers.

The study's inclusion criteria do not consider socio-professional, age, or gender factors as the study is not representative of a specific population. Primarily, geographical criteria are used to include volunteers based on their proximity to terrorist attacks. Special attention is given to volunteers' statements to verify the coherence of their claims (such as their presence at the scenes of the attacks) without delving into specific details. Non-Inclusion Criteria are (1) Residents of unaffected cities in different regions (Type C4). (2) Minors. (3) Volunteers who do not wish to be filmed (4) Those who reject the filming authorization contract or do not give their informed consent. All 934 people recruited were filmed during the interview.

The investigators were carefully selected and were typically individuals with doctorate degrees or doctoral candidates, specializing in fields such as sociology, anthropology, and psychology. They possess prior experience in conducting interviews and fieldwork, particularly in challenging and sensitive contexts. To ensure inclusivity, recruitment efforts also focus on diversifying the age range of the investigators. The training process is extensive and spans several days. It includes,

1. Program Orientation: Investigators receive comprehensive explanations and presentations covering all scientific dimensions of the program.
2. Test Interviews: Training involves conducting test interviews both in a controlled studio environment and in real-world field settings.
3. Specialized Training: Investigators receive specific training for engaging with individuals from the first circle. This training is conducted through collaborative sessions with specialists in post-traumatic stress disorders, who have contributed to the development of the interview guide.
4. Utilization of Tools: Investigators are equipped with two key resources: an interview guide for questionnaires (comprising 28 pages) and an investigator's manual (12 pages). These resources provide detailed instructions on how to handle follow-up interactions and maintain the appropriate behavior while conducting interviews.

# Supplementary Figures

### **Figure S1: Description of the process to build the Research Dataset**

A1, A2, A3, and A4 represent the exposition criterion of DSM-5


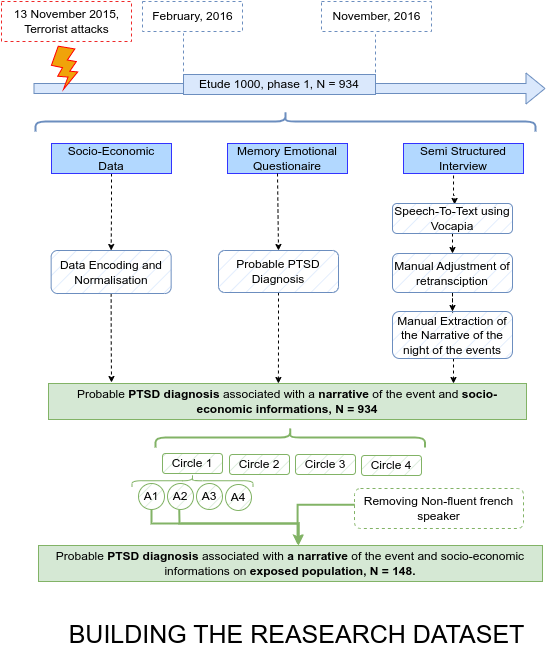


##

### Figure S2. Processing for computing Graph Languages Features


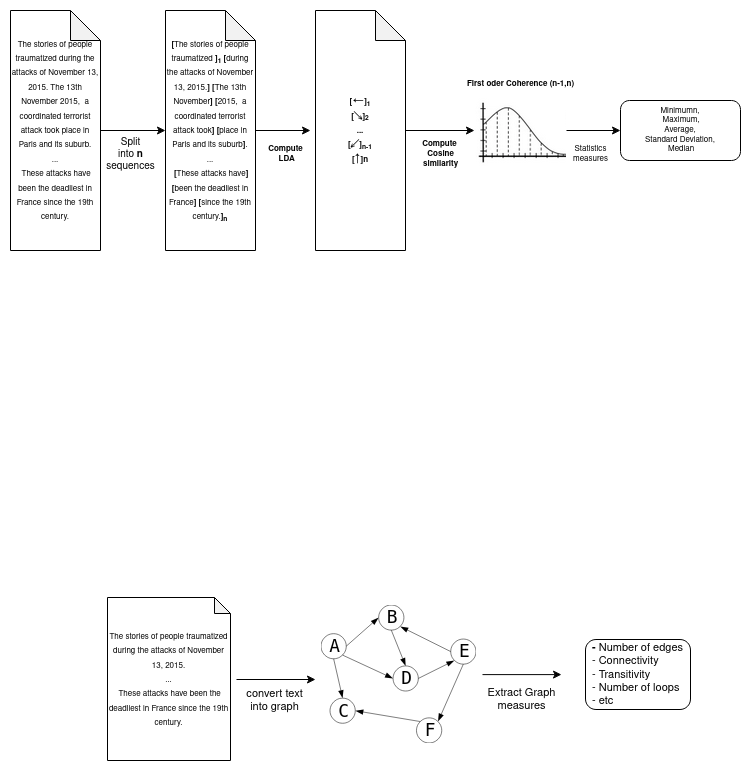


### Figure S3: Multi-channel Classifier from Vanni et al. 2023.

Three channels of convolution/deconvolution for three representations of the input 1) full-forms (words), 2) part-of-speech (POS), 3) lemma
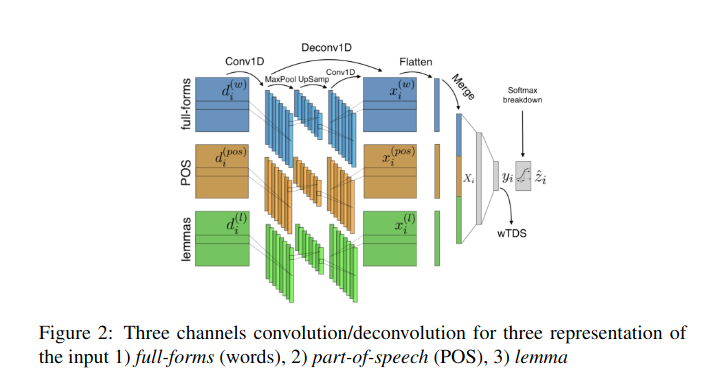


### Figure S4. Distributions of the number of words based on exposition type and PTSD diagnosis
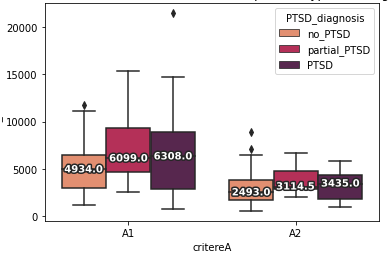


### Figure S5: Distributions of the number of words based on gender and PTSD diagnosis
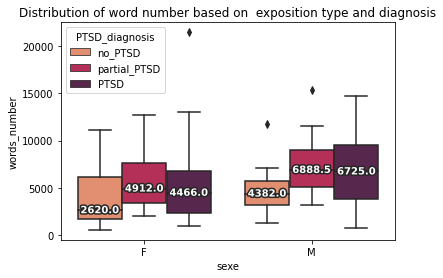


### Figure S6: Description of multiple evaluation metrics for ML Classifiers

Performances on the train and test set averaged over 100 random runs
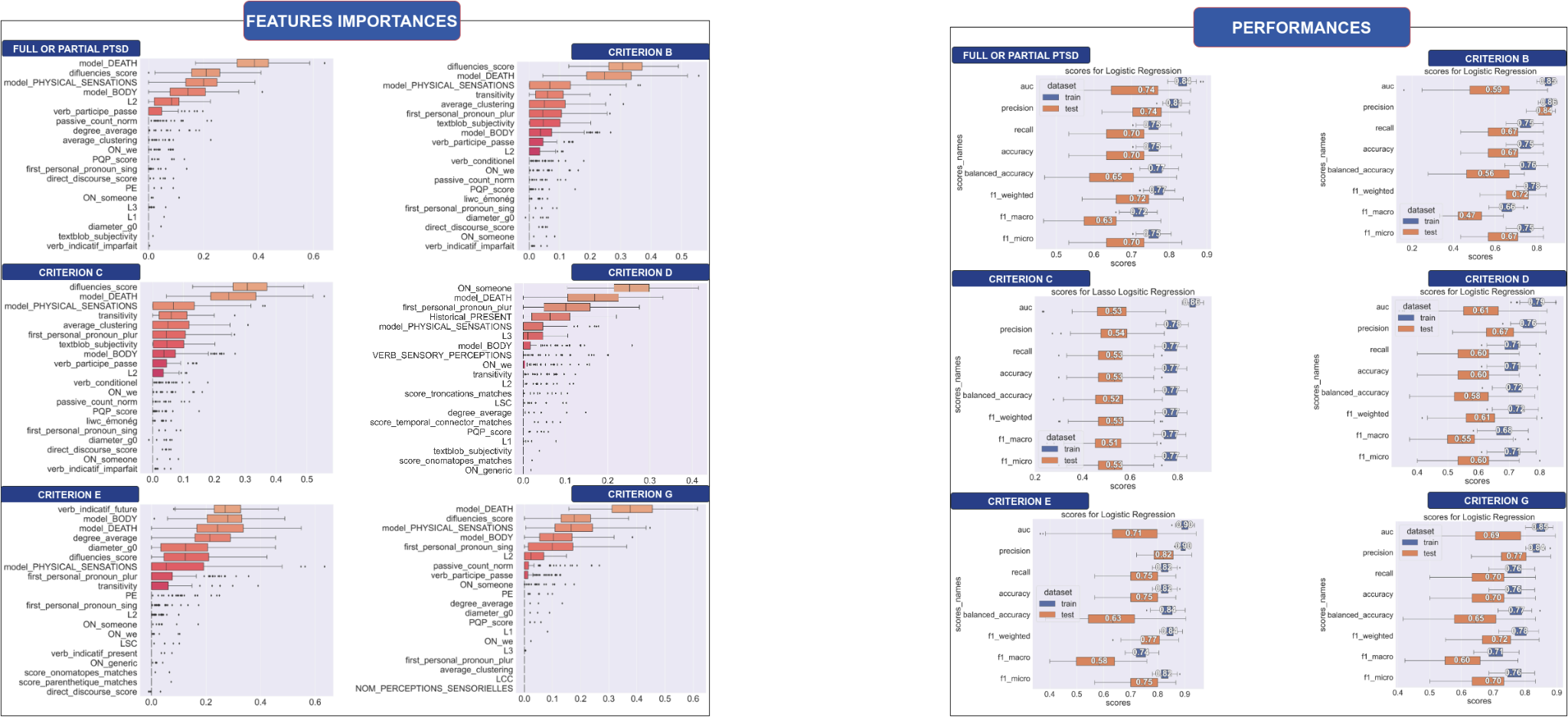


### **Figure S7.** Average ROC curves for each text CNN Classifier related to each DSM-5 Criterion
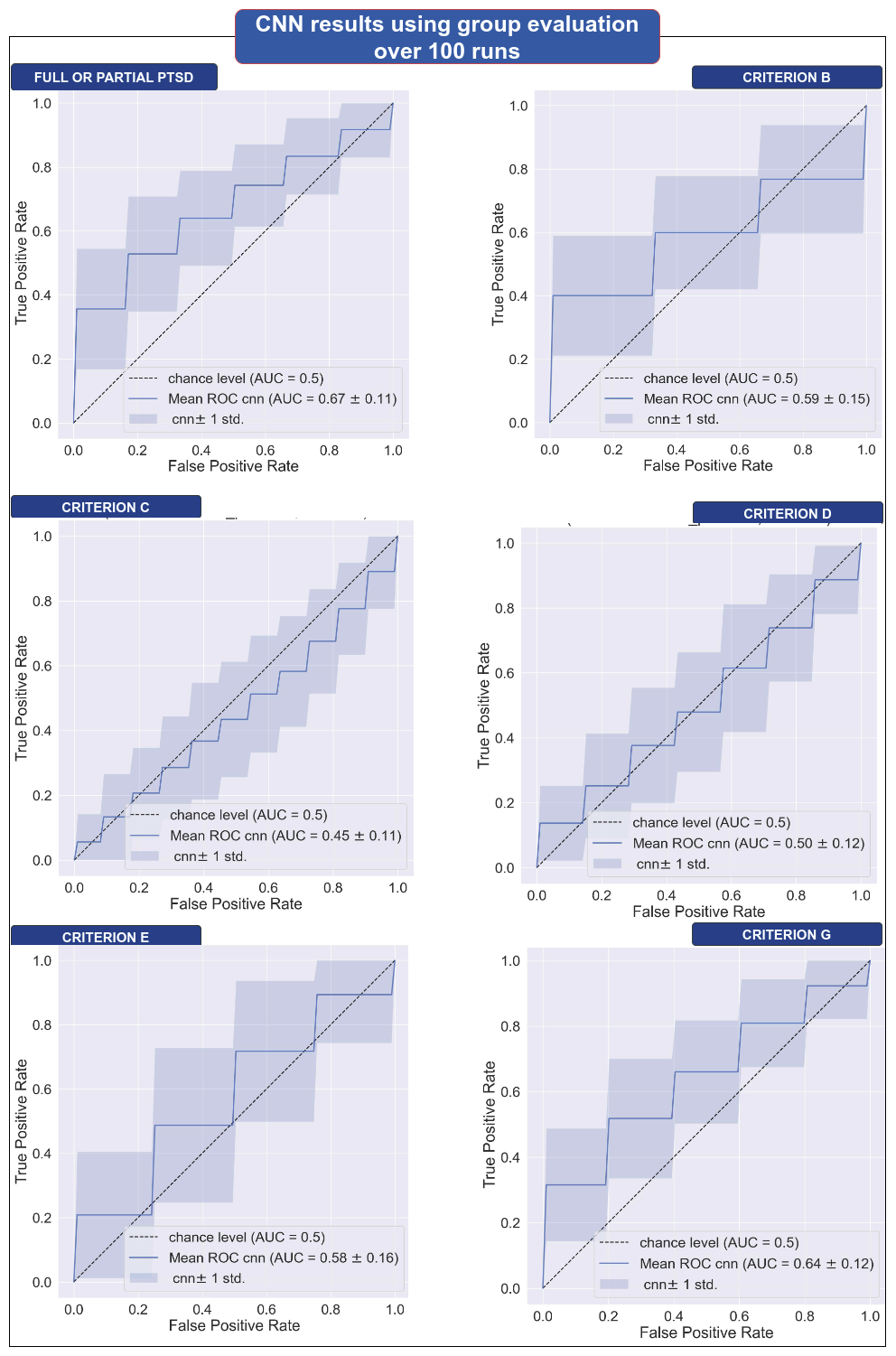


### Figure S8. Average ROC curves for each text CNN Classifier related to each DSM-5 Criterion **using sequences-based evaluations**
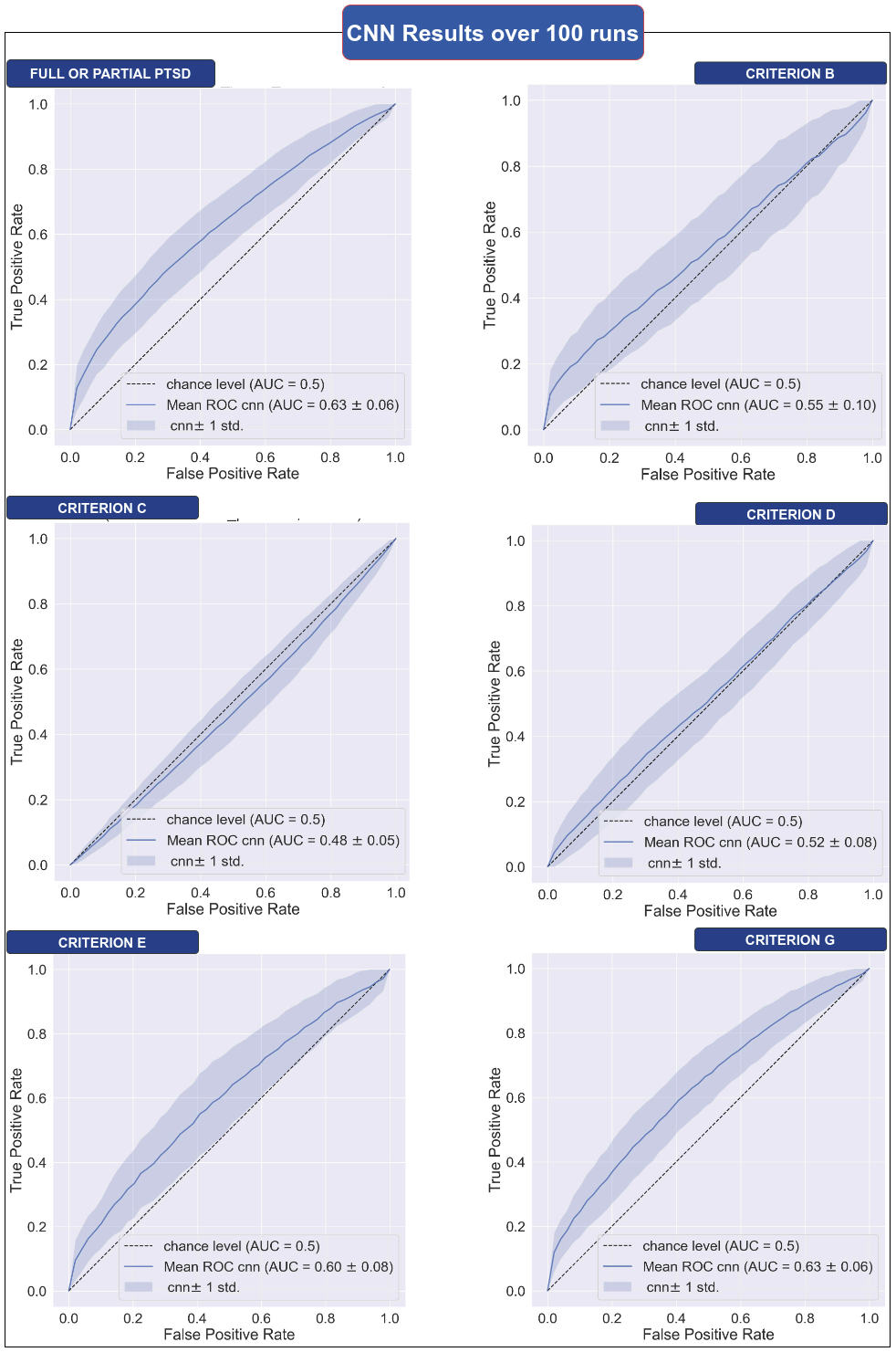


# Supplementary Tables

### Table S1. - Exhaustive list and description of language features

| **Feature Name** | **Description** | **N** |
| --- | --- | --- |
| xxxx_number | Number of words or sentences in each narrative | 2 |
| **Sentiments and Emotions** Features | | **27** |
| liwc_xxx | Proportion of words associated with the xxx’s **LIWC** French category. We selected only the categories associated with emotions, death and body | 4 |
| gobin_xxx | Emotional valences of 10 emotions (disgust, sadness, fear etc.) | 10 |
| textblob_xxx | Polarity and subjectivity scores | 2 |
| labMT | labMT happiness score | 1 |
| feel_xxx | Score associated to 7 emotion, based on **FEEL** lexicon | 7 |
| polarimot_xxx | Score of polarity based on **polarimot** lexicon (neutral, positive, negative) | 3 |
| **Lexical** Features | | **4** |
| model_PERCEPTIONS_xx | Proportion of verb and word related to sensorial perception | 2 |
| model_PHYSICAL_SENSATION | Proportion of token associated with physical sensation | 1 |
| model_DEATH | Proportion of tokens associated with death | 1 |
| **Morphological** features | | **18** |
| xx_personal_pronoun_x | Proportion of personal pronoun for singular and plural of each person (1,2,3) | 4 |
| verb_tense_x | The proportion of each tense in the narrative (past participle, imperfect, present, future, conditional) | 6 |
| PQP_scores | Proportion of verb conjugate to “Plus Que Parfait” tense | 1 |
| modell_ON_xx | Proportion of each value of the pronoun on (generic, someone, we) | 3 |
| model_PRESENT_xx | Proportion of each value of the present (generic, enunciative, historical) | 3 |
| prop_PROPN | Proportion of proper nouns in the text | 1 |
| **Syntactic** Features | | **4** |
| passive_count | The number of passive forms found | 1 |
| passive_sents_count | Number of sentences in passive form | 1 |
| passive_percentages | Proportion of sentences in passive form | 1 |
| passive_count_norm | Passive_count normalized by the logarithm of token numbers | 1 |
| **Disfluency** Features | | **9** |
| repetition_x_order | Normalize count of repetition of syllables, distanced by x syllabus | 3 |
| score_generical_connector_matches | Proportion of connectors in text : ‘mais','donc','aussi','parce que', "et", etc. | 1 |
| score_temporal_connector_matches | Proportions of temporal connectors in text: "quand", "lorsque", "avant que" | 1 |
| score_parenthetique_matches | Proportion of parentheticals in text: "à savoir", "c'est-à-dire" etc. | 1 |
| score_paticules_matches | Proportion of discourse particles in text: "bon","bah","voila","ben","quoi" | 1 |
| score_troncations_matches | Normalized number of “...” in text, that referred to truncated words | 1 |
| score_onomatopea_matches | The proportion of onomatopoeia in text : “crac” etc.. | 1 |
| **Readability Features** | | **5** |
| xx_ratio_score | Lexical diversity score for nouns, adverbs and verbs | 4 |
| direct_discourse_score | Proportion of direct discourse in text | 1 |
| **Discourse coherence** features | | **11** |
| degree_average | Average degrees of each node in the text graph | 1 |
| PE (Parallel Edges) | Number of nodes with a degree superior to 1: number of words repeated more than one time. | 1 |
| average_clustering | Average clustering the text graph | 1 |
| average_shrotest_path_g0 | Average shortest path in the largest connected graph | 1 |
| diameter_g0 | Diameter of the largest connected graph | 1 |
| LCC, LSC | Number of nodes weakly and strongly connected, which is a lexical diversity index | 2 |
| transitivity | Number of triangles in the graph divided by the number of triplets | 1 |
| L1, L2, L3 | Adjacent matrix trace representing the Number of repetition at different order | 3 |
| **Total = 80 Language Features** | | |

### Table S2: Reported performances of detecting passive voice in our corpora using the custom spacy matcher

| Model | Recall | Precision | f1-score |
| --- | --- | --- | --- |
| Spacy large | 0.84 | 0.70 | 0.77 |
| spacy transformer | 0.89 | 0.85 | 0.86 |

### Table S3: Dataset Size and Performances for each custom camembert-based token classifier models

| Task | Labels Name | Dataset size (number of labels) | | | Performances on validation | | |
| --- | --- | --- | --- | --- | --- | --- | --- |
|  |  | Train | Test | Validation | Precision | Recall | f1-score |
| **Value of present tense** | Historical Present | 1131 | 292 | 156 | 0.83 | 0.8 | 0.82 |
|  | Enunciative Present | 2253 | 603 | 324 | 0.80 | 0.83 | 0.82 |
|  | Generic Present | 732 | 192 | 104 | 0.54 | 0.60 | 0.56 |
| **Value of “on” pronoun** | “on” meaning we | 1088 | 310 | 157 | 0.91 | 0.96 | 0.93 |
|  | “on “meaning someone | 102 | 29 | 20 | 1 | 0.8 | 0.89 |
|  | “on” with generic value | 235 | 44 | 16 | 0.64 | 0.56 | 0.60 |
| **Specific lexical fields** | death | 147 | 35 | 18 | 0.93 | 0.83 | 0.88 |
|  | body | 543 | 140 | 98 | 0.90 | 0.87 | 0.88 |
|  | physical sensations | 115 | 27 | 18 | 0.68 | 0.94 | 0.80 |
|  | sensory perception verbs | 636 | 195 | 85 | 0.79 | 0.91 | 0.84 |
|  | sensory perception words | 294 | 102 | 44 | 0.88 | 0.84 | 0.86 |

###

### **Table** S4**.** Metrologic validation of the trauma interview on the 85 participants of the “Etude 1000” who were diagnosed using SCID.

| Diagnosis | Precision | Sensibility | Specificity |
| --- | --- | --- | --- |
| PTSD | 0.95 | 0.92 | 0.96 |
| Partial PTSD | 0.71 | 0.77 | 0.94 |
| Partial PTSD or PTSD | 0.94 | 0.94 | 0.91 |

###

###

### Table S5. Synthesis of the results produced by statistics and machine learning analysis

**🮱 Statistical Evidences 🗹 Machine Learning Evidences ✅ Deep Learning interpretation**

| **Feature Name** | **Full or partial PTSD** | **Criteria** | | | | |
| --- | --- | --- | --- | --- | --- | --- |
|  |  | **B** | **C** | **D** | **E** | **G** |
| Words number | **🮱** |  |  |  | **🮱** | **🮱** |
| sentences_number |  |  |  |  | **🮱** |  |
| **Sentiment and emotional features** | | | | | | |
| labMT | **🮱** |  |  |  | **🮱** | **🮱** |
| feel_positive | **🮱** |  |  |  | **🮱** |  |
| liwc_positive_emotion | **🮱** |  |  |  |  | **🮱** |
| gobin_positive | **🮱** |  |  |  |  | **🮱** |
| gobin_joy | **🮱** |  |  |  |  |  |
| textblob_polarity |  |  |  |  | **🮱** |  |
| textblob_subjectivity | **🗹** | **🗹** |  |  |  |  |
| **Lexical features** | | | | | | |
| BODY | **🮱 🗹** | **🮱🗹** | **🮱 🗹** | **🗹** | **🮱🗹** | **🮱 🗹** |
| PHYSICAL_SENSATIONS | **🮱 🗹** | **🮱 🗹** | **🮱 🗹** | **🗹** | **🮱🗹** | **🮱 🗹** |
| liwc_death | **🮱** | **🮱** |  |  |  | **🮱** |
| DEATH | **🗹 ✅** | **🮱 🗹** |  | **🗹** | **🮱 🗹** | **🮱 🗹** |
| PERCEPTION VERBS | **🗹** | **🗹** | **🗹** | **🗹** | **🗹** |  |
| **Morphosyntactic Features** | | | | | | |
| Past Participle | **🗹** |  | **🮱 🗹** |  |  |  |
| PRON |  |  |  | **🮱** |  |  |
| Proper Name |  |  |  |  | **🮱** |  |
| ON_someone |  |  |  | **🮱 🗹** |  | **🗹** |
| ON_we | **🗹** |  |  |  |  |  |
| first_personal_pronoun_plur |  | **🗹** |  | **🮱 🗹** | **🗹** |  |
| verb_indicatif_future |  |  |  |  | **🮱🗹** |  |
| first_personal_pronoun_sing | **🗹 ✅** |  |  |  | **🗹** | **🮱🗹** |
| Historical_Present | **🗹** |  |  | **🗹** |  | **🗹** |
| Generical_present | **🮱** |  |  |  |  | **🮱** |
| Direct Discourse score | **🗹** |  |  |  |  |  |
| **Syntactic Features** | | | | | | |
| passive_count_norm | **🮱 🗹** |  |  |  | **🮱** | **🮱🗹** |
| **Speech Disfluencies Features** | | | | | | |
| score_troncations_matches | **🮱 🗹 ✅** | **🮱 🗹** |  |  | **🗹** | **🗹** |
| score_generical_connector_matches |  |  |  |  | **🮱** |  |
| **Readability Features** | | | | | | |
| token_ratio_score | **🮱** |  |  |  | **🮱** | **🮱** |
| noun_ratio_score | **🮱** |  |  |  | **🮱** |  |
| adverb_ratio_score |  |  |  |  | **🮱** |  |
| **Discourse Coherence Features** | | | | | | |
| graph_PE | **🮱** |  |  |  | **🮱 🗹** | **🮱🗹** |
| graph_degree_average | **🮱 🗹** |  |  |  | **🮱 🗹** | **🮱** |
| graph_average_clustering | **🮱** | **🗹** |  |  |  |  |
| graph_L2 | **🮱 🗹** | **🗹** |  |  | **🮱🗹** | **🮱🗹** |
| graph_L3 | **🮱 🗹** |  |  | **🗹** |  |  |
| graph_average_shrotest_path_g0 |  |  |  |  | **🮱** |  |
| Diameter_g0 | **🗹** |  |  |  | **🗹** |  |
| graph_transitivity |  | **🗹** |  |  | **🗹** |  |

###

### Table S6. Not Translated most significant sequence associated with Full or Partial PTSD

Extracted using CNN probabilities

| [...] un rapport à la mort , dans ces cas là ... où ben j' en avais vu d' autres de les morts ... dans le Bataclan ... et euh ... de le coup bon moment OK ... Marie est morte , et c' était plus par rapport à sa famille , où je penser à sa famille , me dire : " C' est trop con , elle est arrivée jusque -là et ... ben , c' est à le poste de secours , où elle est décédée alors que c' est le ... voilà . " Don c' est ... mais je me suis pas ... je me suis pas de le tout projetée par contre à me dire que ça pourrait être moi ... ça , c' est des mois après que j' ai pu me dire des choses comme ça , mais ... pas de le tout sur le coup . Euh ... de le coup euh ... bah voilà Marie est décédée et puis ... dans ... c' était très fouillis , je me souviens que les secours ... on sentait qu' y avait quand même un peu de panique quoi , c' est ... je les voyais s' agiter mais revenir quinze fois à le même endroit et la pauvre Marie on est venu à le moins trois , quatre fois , voir si elle allait bien et puis elle était décédée à chaque fois donc euh ... donc PRON , je , je me souviens , de m' être dit aussi que , normalement , on fait des codes couleurs pour ça , pour que ... pour identifier les , les , les , décédés , les ... l' état de gravité de les blessés . Donc euh ... donc voilà . Et euh ... et à le moment où j' ai commencé à vraiment trouver le temps long , je me suis ... je me souviens avoir croisé le regard ... je me suis peut -être relevée , un peu ... et j' ai croisé le regard d' une fille qu' on était en train d' évacuer , et je me souviens qu' on s' est regardé et que c' était vraiment ... une connivence , de se dire: " Putain mais de quoi on ressort là ? " De voir ... oui , le regard comme on pouvait pas se parler , parce qu' on était à l' autre bout de la cour , l' une de l' autre , mais vraiment de ... et puis contente d' accrocher le regard de quelqu'un , aussi . Donc cette fille est partie et puis on a quand même fini par m' évacuer , mais j' étais ... y avait plus grand monde dans la cour , quand on m'[..] |
| --- |

# Interview Material (Translated)

## First Part, open questions about the event

[See the Manual for the preparation in front of the camera and before the interview]

*First of all, thank you very much for accepting this interview.*

*We are going to start with a filmed exchange, during which I will ask you to look at me, rather than at the camera. During this exchange, I may take some written notes: don't worry about it, it simply helps me to follow what you are saying.*

*This interview will take place in two stages: a first part that is fairly free, and a second during which I will ask you a few more precise questions, requiring short answers. Then we will stop the video recording, and I will accompany you to another room where you will fill in a short written questionnaire.*

QUESTION 1

**«*To begin with, could you tell me about November 13^th^ 2015?»***

The interviewee can spontaneously interpret the question as he or she wishes. Thus, if he/she asks: «*What happened or what I experienced?*»

Answer: «*As you wish.*»

- **1st scenario:** he starts with his personal experience. Let his speech develop by encouraging him (cf. below) and, after the thematic relaunches, open with the account of the events if he does not address it spontaneously.
- **2nd scenario:** he starts with the account of events ("what happened"). Leave the speech develop by encouraging him (cf. below) and, after the thematic prompts, move on to the personal experience if he does not address it spontaneously.

Personal experience of November 13th

Do not forget to encourage the interviewee to expand on what he or she has said as much as possible **by encouraging them to say more, by repeating a word or phrase from the volunteer if the person is silent.**

##

## Second Part, Semi-standardized interview for PTSD diagnosis assessment

*«I'll start by asking you some questions about your memories.»*

**QUESTION 1.**

**First, do you feel like you've been thinking about November 13 and the days that followed in this past month?**

o No:

o If so, please specify: «often", «too often» or neither"?

**QUESTION 2.**

**Have you ever had images or thoughts related to these events come back to you unexpectedly?**

o No

o Yes

**Do you ever think about it deliberately, in the form of thoughts or general memories?**

o No

o If yes:

**2.1.1. Are these thoughts distressing?**

o No: how would you characterize them? ……………………………………………….

o If so, please specify: «very distressing» or «a bit distressing» (mark the correct answer)

**2.1.2. How often would you say you have thought about this in the past month?**

Please specify: «not at all», «several times a week», «once a week», «several times a month», «once a month»

**2.1.3. Do you feel that you have been dwelling on this event?** o No

o If yes: again this last month? ………………..

If yes: how often this last month?: «several times a week»,»once a week», «several times a month», «once a month»

is it rather in the form of:

Sensations (or precise images)

Thoughts (i.e. general memories or reflections)

Both

DK

**What form do they take?**

Specify: «sounds», «images», «odors», «tactile sensations»

**Do they sometimes seem so vivid or detailed that you feel as if you are (re)experiencing the scene?**

o No

o Yes

**Are they distressing?**

o No: how would you characterize them? ……………………………

o If yes, please specify: «very distressing», «a little distressing»

**Did these specific memories occur again this last month?**

o No

o If so, please specify their frequency: «several times a week»,»once a week», «several times a month», «once a month»

**Did you also deliberately think about it this past month?**

o No

o If so, please specify their frequency: «several times a week», «once a week», «several times a month», «once a month»

o **Do you have the impression that you have, at some point, dwelled on this event?**

o No

o If so: still the case this last month?

If so: how often this last month? «several times a week», «once a week», «several times a month», «once a month»

**Are these thoughts distressing?**

o No: how would you characterize them? ………………………….

o If so, please specify: very distressing, a little distressing

**Have these thoughts occurred again this past month?**

o No

o If so, please specify their occurrence: «several times a week», «once a week», «several times a month», «once a month»

**Did you ever think about it deliberately?**

o No

o If so, please specify the frequency: «several times a week», «once a week», several times a month», «once a month»

**Do you feel that you have been rehashing this event?**

o No

o If so: also this last month?

If so: how often this last month? «several times a week», «once a week», «several times a month», «once a month»

**When you think about it, is it things that you have experienced, media images or things that you have been told?**

*If nothing matches, add other*:

*If an example is given spontaneously, note it:*

**QUESTION 3.**

**Do you ever try to avoid thinking about it?**

o No

o If so: What do you do about it?

…………………………………………………………….

**QUESTION 4.**

**During this last month, have you ever returned to or avoided certain locations, certain media, certain people?**

o No

o If so, please specify:

Certain locations?

Returned to: No Yes: specify………………. Avoided No Yes: specify …………….

Certain media?

Returned to: No Yes: specify ……………..

Avoided: No Yes: specify ……………..

Certain people?

Returned to: No Yes: specify ………………. Avoided:

No Yes: specify ……………….

**QUESTION 5.**

**What emotions do you feel when you think about the events of November 13 during this past month?**

Feelings: *free expression* …………………………………………………………..

Then *propose*: anger, incomprehension, empathy, sadness, disgust, fear, astonishment, surprise, interest, joy, satisfaction...

**QUESTION 6.**

**Does thinking about these events trigger any physical reactions in you?**

o No

o If so (*or hesitation*), suggest: trembling, pain, sweat, difficulty to breathe, stomach ache, palpitations...

If so: does it still happen this last month?

**QUESTION 7.**

**Do events ever come back to you in your sleep?**

o No

o if so:

Dreams or nightmares? (mark the answer)

Very close to reality? Yes No

Was it still true this last month? Yes No

■ if so: How often? ………………………………….

**Have you had any event-related difficulties in the past month:**

To fall asleep? Yes No

Waking with a start ? Yes No

Other sleep related problems? Yes No

*«We are now going to ask you about things that may have changed for you since November 13 and the events that followed.»*

**QUESTION 8.**

**Would you say that you have confidence in the media's information about November 13th?**

o Rather Yes

o Rather No

o Not at all or feeling of manipulation

**QUESTION 9.**

**Could you tell us if this event has changed the way you function in your daily life or in your relationships?**

o No

o If so, please specify: Work, studies, family, couple, friends?

(Complete the following only in the area(s) where changes are reported)

Are these changes:

Work or studies:

More positive or negative? Intense or moderate?

Do they still persist this last month?

Family or couple:

More positive or negative? Intense or moderate?

Do they still persist this last month?

Friends:

More positive or negative? Intense or moderate?

Do they still persist this last month?

**QUESTION 10.**

**Do you feel that you have matured, or that you think better about the world or about your life since these events?**

About the world:

o No

o Yes

About your life:

o No

o Yes

**QUESTION 11.**

**Are you sometimes more on guard since these events?** Yes No

**Do you have startle reactions?** Yes No

**Do you take any special precautions?** Yes No

If yes: specify………………………………………………

**Do you feel more irritable?** Yes No

**QUESTION 12.**

**Do you forget things more than usual?**

o No

o if so: can you give an example ………………………………..

**Have you ever felt on** «**autopilot», i.e. disconnected from reality??**

o No

o If so: more than you used to?

if so: is it still the case this last month?

o No

o Yes

**Do you have difficulty concentrating?**

o No

o If so: more than you used to?

if so: still during this last month?

o No

o Yes

**Are you particularly distracted?**

o No

o Yes

o If so: more than you used to?

if Yes: Still during this last month?

o No

o Yes

**QUESTION 13.**

**Do you often talk about these events?**

o No

o If so, specify: To whom? …………………

*Free expression*

Then *proposal:* Friends, family, colleagues, health care professionals …

How often?.......................

Do you talk more about events in general or memories that come back to you?

**QUESTION 14.**

**Has your involvement in certain activities changed since?**

o No

o if so, which activity?

*Propose* Political activities, Cultural activities, Leisure and entertainment activities, Religious activities, Professional activities …

o For each activity mentioned, specify: s’agit-il d’une implication plus forte ou au contraire de moins d’engagement, de moins d’intérêt?

**QUESTION 15.**

**Overall, do you feel more optimistic or more pessimistic?**

*Both answers are possible*

**In relation to the world as it is?**

o More optimistic

o More pessimistic

o Neither of them

If yes to any of these questions: Would you say a little or a lot?

**What about yourself, your qualities or your future?**

o More optimistic

o More pessimistic

o Neither of them

If yes to any of these questions: Would you say a little or a lot?

**QUESTION 16.**

**Finally, and this will be my last question, did the events of November 13 remind you of other events?**

**From your personal life?**

o No

o If so, please specify which ones:

**From your family?**

o No

o If so, please specify which ones:

**Or even History (with a capital H)?**

O No

o If so, please specify which ones:

# Experimental Report

In this section, we will present the experiments we made during our research process. This section aims to aliment the curiosity of researchers working on the same problem as ourselves and provide richer insight to create new experiments. In our study, we are working on 6 classification text problems, one for each symptom and 1 for the complete diagnosis. For the sake of clarity and shortness, we will present experiments only on the classification related to “full or partial PTSD” diagnosis.

## Machine Learning Experiments

**Question:** Do the emotional features (Gobin, Plarimot FEEL) greatly influence the results?

**Observations:** The emotional features do have a significative importance concerning classification performances. Figure E1 presents the AUC scores over 100 runs, (a) with the 12 emotional features related to emotional valence ( including Polarimots and Gobin); (b) with all 27 emotional features.
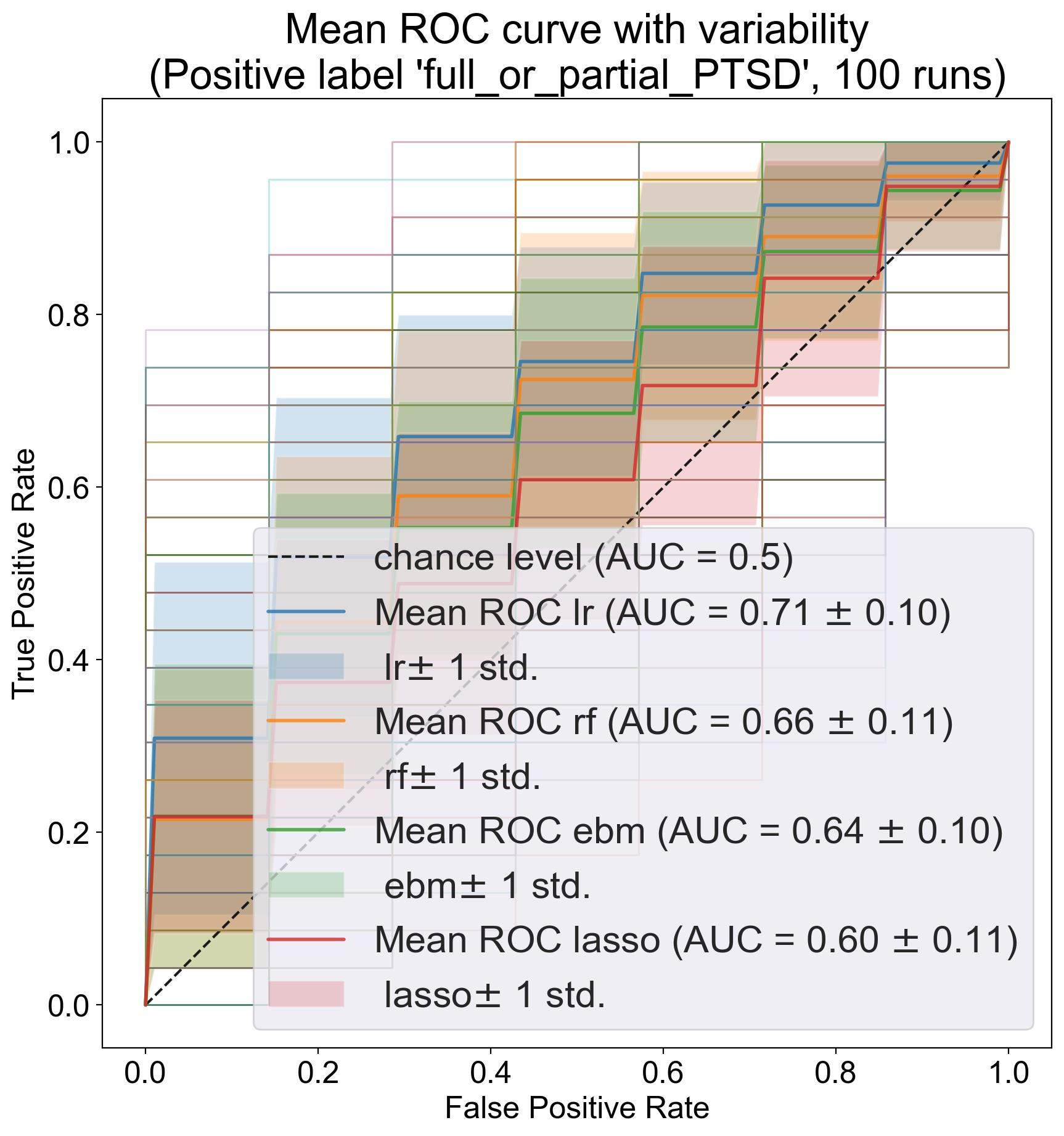

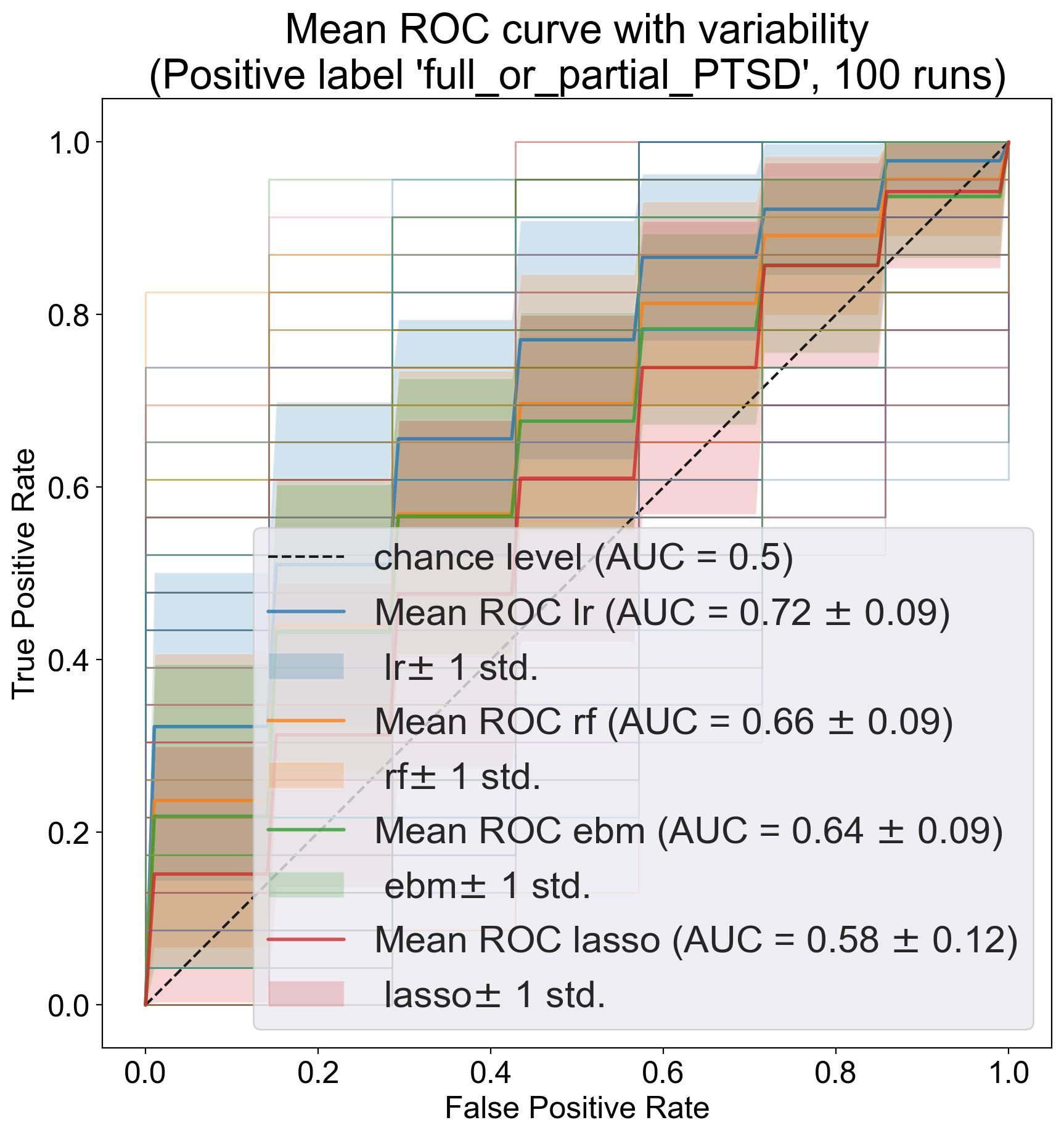


*Figure E1: AUC scores over 100 runs, (on the left) with the 12 emotional features related to emotional valence ( including Polarimots and Gobin); (on the right) with all the 27 emotional features (including categorical features).*

**Question:** Does data augmentation have a significant impact on classification performance?

**Observation:**  As our dataset is small, we considered using SMOTE on our dataset before using the classification algorithm. We found that the SMOTE algorithm does not improve nor impact our performance (AUC = 0.68 ± 0.1). This aligns with the recent paper from [Elor et Averbuch-Elor (2022)](https://www.zotero.org/google-docs/?pST0FK) that mitigates the positive effect of SMOTE on an imbalanced dataset.

**Question:** How can we find the best hyperparameters set for Machine Learning models?

**Exploring the Hyper-parameter space for ML classifier:** we select the parameter for Random Forest and Logistic Regression by performing a grid-search optimization algorithm using the hyperOpt ([Hyperopt Documentation](http://hyperopt.github.io/hyperopt/)) python library. The list of hyperparameter spaces is reported in Table E2. For each model and classification problem, we found the optimal parameter and associated score (presented in Table E3).

Table E1: Hyperparameter Search Space Overview for Machine Learning Algorithms

| Random Forest | n_estimators | Discrete Uniform [5, 50] |
| --- | --- | --- |
|  | max_depth | Discrete Uniform [5, 25] |
|  | min_samples_split | Continuous Uniform [0, 1] |
|  | min_samples_leaf | Discrete Uniform [1, 20] |
|  | criterion | {'gini', 'entropy'} |
|  | max_features | {'sqrt', 'log2'} |
|  | class_weight | {'balanced', {0:1,1:5}, {0:1,1:10}, None} |
| Logistic Regression | penalty | {'l1', 'l2', 'elasticnet', 'none'} |
|  | C | Continuous Uniform [0, 5] |
|  | fit_intercept | {True, False} |
|  | l1_ratio | Continuous Uniform [0, 1] |
|  | solver | {'saga'} |
|  | class_weight | {'balanced', {0:1,1:5}, {0:1,1:10}} |
| Explainable Boosting Machine | max_bins | Discrete Uniform [5, 50] |
|  | max_rounds | {100, 200, 300} |
|  | binning | {'uniform', 'quantile'} |
|  | learning_rate | Continuous Uniform [0.0001, 0.01] |
|  | interactions | {0, 5, 10} |
|  | min_samples_leaf | Discrete Uniform [2, 30] |
|  | max_leaves | Discrete Uniform [2, 10] |
|  | early_stopping_rounds | Discrete Uniform [10, 50] |

Table E2: Scores and best parameters for each classification problem

| Classification task | models | AUC Scores (average 100) | Best parameters for best models |
| --- | --- | --- | --- |
| Full or Partial PTSD | Random Forest | 0.66 | {C =0.1, class_weight =”balanced”,l1_ratio = 0.69, penalty = “elasticnet”, solver = “saga”} |
|  | **Logistic Regression** | **0.72** |  |
|  | Explainable Boosting Machine | 0.64 |  |
| Criterion B  (Intrusion) | Random Forest | 0.55 | {C = 4, class_weight =”balanced”,l1_ratio = 0.03, penalty = “elasticnet”, solver = “saga”} |
|  | **Logistic Regression** | **0.66** |  |
|  | Explainable Boosting Machine | 0.54 |  |
| Criterion C  (Avoidance) | Random Forest | 0.55 | {C = 3.1, class_weight =”balanced”,l1_ratio = 0.18, penalty = “none”, solver = “saga”} |
|  | **Logistic Regression** | **0.56** |  |
|  | Explainable Boosting Machine | 0.55 |  |
| Criterion D (Dissociation) | Random Forest | 0.61 | {C =2.7, class_weight =”balanced”, penalty = “l1”, solver = “saga”} |
|  | **Logistic Regression** | **0.69** |  |
|  | Explainable Boosting Machine | 0.62 |  |
| Criterion E  (HyperArousal) | Random Forest | 0.77 | {C = 2.9, class_weight =”balanced”,l1_ratio = 0.3, penalty = “none”, solver = “saga”} |
|  | **Logistic Regression** | **0.78** |  |
|  | Explainable boosting Machine | 0.77 |  |
| Criterion G  (Significant distress) | Random Forest | 0.69 | {C = 0.01, class_weight =”balanced”,l1_ratio = 0.3, penalty = “l2”, solver = “saga”} |
|  | **Logistic Regression** | **0.73** |  |
|  | Explainable boosting Machine | 0.68 |  |

## Deep Learning Experiments

**Question:** Does the model architecture greatly influence classification performances?

**Experiment:**  We compute the average performance scores (group and ungroup) for different model architectures on 15 random seeds. The results may vary from those presented on the paper which is averaged on 100 different seeds. These results are not a benchmark but could serve other researchers who can benefit from them to start their research. All the computations were made on a local GPU Nvidia RTX A 4000, using tensorflow 2.12.0.

**Observation:**  Based on the results presented in Table E3, the CNN classifier seems to be the best compromise between the number of parameters and the performances on our dataset. This observation is not proof that CNN is better for text classification, this is solely some data to justify our use of CNN. We do not have the computed capacity to propose a complete benchmark and grid search algorithm over all the models' parameters.

Table E3. Report of different AUC scores over 15 runs depending on model architecture. (*mean* ± std)

| Model name | Architecture or weights | Number of parameters | Sequence size | AUC (average 15) | Grouped AUC (average 15) |
| --- | --- | --- | --- | --- | --- |
| Keras - CNN | Embedding (128) SpatialDropout1D  Convolution1D  AveragePooling  Dense  Softmax | ~1.8 M | 512 | **0.64 ± 0.08** | 0.65 ± 0.13 |
| Keras - LSTM | Embedding (128) SpatialDropout1D  LSTM (128)  LSTM (24)  Dropout  Dense  Softmax | ~0.5 M | 512 | 0.56 ± 0.12 | 0.59 ± 0.13 |
| Keras - Attention | Embedding (128)  Bidirectional LSTM (128)  Bidirectional LSTM (64)  Concatenate  Attention (10)  Dense  Softmax | ~0.5 M | 512 | 0.57 ± 0.11 | 0.60 ± 0.14 |
| Keras - Transformers | <https://keras.io/examples/nlp/text_classification_with_transformer/> | ~0.5 M | 512 | 0.58 ± 0.07 | 0.61 ± 0.11 |
| CambertBase | [almanach/camembert-base · Hugging Face](https://huggingface.co/almanach/camembert-base) | ~110 M | 512 | 0.62 ± 0.05 | **0.69 ± 0.09** |
| Distilbert muti cased | [distilbert \| Kaggle](https://tfhub.dev/jeongukjae/distilbert_multi_cased_preprocess/2) | ~134 M | 512 | 0.63 ± 0.07 | **0.69 ± 0.12** |

**Question:** Does the Focal Loss have an impact on the CNN classifier?

**Experiment:**  We reproduce the paper results by averaging 100 runs of CNN classification over random seeds and we compare with and without the use of Focal Loss. The gamma parameter is equal to 2 as suggested by [Lin et al. (2018)](https://www.zotero.org/google-docs/?FEMamp) and the alpha parameter is fixed at 0.7.

**Observation:**  As reported in Table E4, the focal loss has a positive impact on the performances of the CNN classifier. We hypothesize that the difficulty in differentiating PTSD, that means the expression of PTSD, using markers in the language varies across the testimonies. The gamma parameter helps the model to concentrate on the “difficult” example and avoid overlearning the simple one.

*Table E4: AUC scores over 100 runs, (on the left) with the use of Focal Loss as presented in the paper, α =0.7 γ = 2; (in the middle) with the use of only the alpha parameter (α =0.7 γ = 0); and (on the right) the classical cross-entropy loss (α =0.5 γ = 0) (mean* ± std)

| Model name | Focal Loss Parameter | AUC (average 100) | Grouped AUC  (average 100) |
| --- | --- | --- | --- |
| Keras_ CNN | α =0.7 γ = 2 | 0.64 ± 0.09 | 0.68 ± 0.1 |
| Keras_ CNN | α =0.7 γ = 0 | 0.63 ± 0.08 | 0.66 ± 0.1 |
| Keras_ CNN | α =0.5 γ = 0 | 0.61± 0.09 | 0.63 ± 0.14 |

## LLM experiments

In recent years, advancements in natural language processing (NLP) have been largely driven by the development of LLMs, which have demonstrated remarkable capabilities in various language-related tasks. By including an experiment utilizing an LLM, we aim to share some intuition on how LLM performs on our task and what work should be done to use them as a reliable tool to classify PTSD and analyze testimonies from survivors. In a recent paper, [Yang et al. (2023)](https://www.zotero.org/google-docs/?DUCcCT) propose to use LLM to detect evidence of mental health conditions on social media posts and generate explanations for the predictions. In these short experiments, we use a quantized version of Mistral 7B [(Jiang et al. 2023)](https://www.zotero.org/google-docs/?mModQX) with a Chain of Thought (CoT) prompts to classify our testimonies. The model (<https://ollama.com/library/neural-chat>) and post-processing have all been done on local GPU (Nvidia A 4000, 8Gb of VRAM).

**Experiment:** We conduct two experiments of prompting classification on our dataset. The first one is without provided examples and the second is with one typical example of each class (with and without Full or Partial PTSD). The two examples were selected by a clinician psychiatrist.

System prompt: *“You are a psychiatrist with expertise in PTSD. You have received a testimonial from someone who has experienced a traumatic event. Your task is to analyze the testimony and classify it as PTSD or Non-PTSD.*

*As a reminder, here's the definition of PTSD:*

*PTSD (Post-Traumatic Stress Disorder) is a mental health disorder that can develop after experiencing or witnessing a traumatic event.*

*Symptoms can include flashbacks, nightmares, severe anxiety, and uncontrollable thoughts related to the event. The disorder can significantly affect daily functioning and quality of life if left untreated.”*

CoT prompt: *“ <<< Testimony to analyze: {testimony} >>>*

*[INST]*

*To detect PTSD, follow these steps:*

*1- Summarize the Testimonial in 100 words:*

*Summarize the testimony in 100 words. Remember the first and last names of the people mentioned.*

*2- Risk Factor Analysis:*

*- Has the person been exposed to death, injured or lost a loved one?*

*- Is there any mention of past trauma?*

*- Was the person active during the event? If so, this is a protective factor.*

*List of risk factors: []*

*3- Direct evocation of symptoms:*

*- Look for descriptions of intrusive thoughts, memories or flashbacks.*

*- Note avoidance behaviors and signs of hypervigilance.*

*- Identify manifestations of negative mood and cognitive changes.*

*List evocations of symptoms present in the narrative: []*

*4 - Direct evocation of treatment:*

*- Explore mentions of seeking professional help, therapy or medication.*

*List of evocations of treatment: []*

*5- Analysis between the lines:*

*- Study the coherence of the speech.*

*- Look for fluidity of speech, interruptions or hesitations (marked by "...").*

*- Note the use of present tense and vivid descriptions.*

*- Identify figures of speech, metaphors or euphemisms.*

*- Look for signs of dissociation and persistent negative emotions.*

*List of relevant verbatims : []*

*6- Conclusion:*

*Include a conclusion based on the analysis as a whole, classifying the testimony as PTSD, or Non-PTSD.*

*The diagnosis must be justified by concrete elements of the testimony and placed between #diagnosis#.*

*[\INST]*

*Analysis:”*

Example Prompt: *“####*

*Here are some examples of testimonial analysis to help you understand how to analyze testimonials.*

*## Example 1*

*{pos_analysis}*

*## Example 2*

*{neg_analysis}*

*####*

**Observations:** The classification performances are detailed in Table E5, and show a notably low level of performance compared to the supervised methods used in our paper. These results raise several considerations. First, the suboptimal nature of our prompt could potentially be a limiting factor, warranting further optimization efforts. Second, the inherent challenges of zero-shot or few-shot learning may contribute significantly to the observed differences when compared to supervised learning methods. Third, it's important to acknowledge the complexity of our classification task compared to typical applications of LLMs in text classification, such as sentiment analysis or social media content categorization. The uniqueness of our dataset lies in its homogeneity; all individuals in the dataset have experienced a deeply traumatic event, and thus consistently express negative sentiments and discuss mortality issues. While this observation is not conclusive, it suggests that considerable refinement is required to effectively adapt LLMs to our classification goals. Finally, the presence of long documents in our dataset may exacerbate problems associated with LLM hallucination, further complicating the classification process. Taken together, these factors highlight the nuanced challenges of using LLMs for our specific task, which requires careful consideration and potentially novel methodological approaches.

*Table E5: AUC scores using mistral-chat*

| Prompt | model | AUC score | F1 score |
| --- | --- | --- | --- |
| CoT prompt | [neural-chat](https://ollama.com/library/neural-chat) | 0.51 | 0.62 |
| Cot prompt with 2 examples | [neural-chat](https://ollama.com/library/neural-chat) | 0.48 | 0.45 |

Example of LLM output for a true positive (uuid= 6e40e574-d41f-549e-a68c-b66dfbb97062) :

*“ 1- Summarize the Testimonial in 100 words:*

*A man recounts his experience during a terrorist attack, describing how he witnessed the death of his friend and the aftermath. He was left traumatized by the event, struggling to cope with the loss and guilt while trying to support his family.*

*2- Risk Factor Analysis:*

*- Has the person been exposed to death, injured, or lost a loved one? Yes, he witnessed the death of his friend CXXX.*

*- Is there any mention of past trauma? No, but he was affected by the event and its consequences.*

*- Was the person active during the event? Yes, he was actively involved in the situation.*

*List of risk factors: [Exposure to death, loss of a loved one]*

*3- Direct evocation of symptoms:*

*- Look for descriptions of intrusive thoughts, memories, or flashbacks. He recalls the details of the attack and his friend's death.*

*- Note avoidance behaviors and signs of hypervigilance. He mentions how he tried to hide from the police and was in a state of shock.*

*- Identify manifestations of negative mood and cognitive changes. He describes feeling numb, unable to process what happened, and being overwhelmed by guilt.*

*List evocations of symptoms present in the narrative: [Intrusive thoughts, hypervigilance, avoidance behaviors, negative mood, cognitive changes]*

*4- Direct evocation of treatment:*

*- Explore mentions of seeking professional help, therapy, or medication. He did not mention any specific treatment.*

*List of evocations of treatment: []*

*5- Analysis between the lines:*

*- Study the coherence of the speech. The narrative is disjointed and difficult to follow, with frequent interruptions and hesitations.*

*- Look for fluidity of speech, interruptions, or hesitations (marked by "..."). He mentions being in a state of shock and unable to process what happened.*

*- Note the use of present tense and vivid descriptions. The man recalls the event in detail, using vivid language.*

*- Identify figures of speech, metaphors, or euphemisms. He uses metaphors like "hallucinant" to describe his experience.*

*- Look for signs of dissociation and persistent negative emotions. He describes feeling numb and unable to process what happened.*

*List of relevant verbatims: [Hallucinating, shock, numbness]*

*6- Conclusion:*

*The diagnosis is PTSD. The man was exposed to a traumatic event involving death and loss, which led him to experience intrusive thoughts, hypervigilance, avoidance behaviors, negative mood, cognitive changes, and dissociation. He also struggled with guilt and the aftermath of the attack, affecting his ability to support his family.*

Example of LLM output for a false negative (uuid: b223a90e-4aec-51ad-aa5b-47c1cd28d564):

*“1- Summarize the Testimonial in 100 words*

*This testimony describes a harrowing experience during a terrorist attack, where the narrator and their friends were caught in the crossfire. They recount the events of that night, from being trapped in a bar to escaping and finding safety with family members. The narrator's account is filled with vivid details, intrusive thoughts, and flashbacks, as well as signs of hypervigilance and avoidance behaviors. There are also indications of negative mood and cognitive changes.*

*2-Risk Factor Analysis:*

*- Has the person been exposed to death, injured or lost a loved one? Yes, there is mention of people being killed and wounded.*

*- Is there any mention of past trauma? No specific past traumas were mentioned.*

*- Was the person active during the event? If so, this is a protective factor. The narrator was actively involved in trying to escape and find safety.*

*List of risk factors: [Exposure to death, injury or loss of loved ones]*

*3-Direct evocation of symptoms: []*

*List of evocations of symptoms present in the narrative: []*

*4- Analysis between the lines:*

*- Study the coherence of the speech. The narrator's account is coherent and detailed.*

*- Look for fluidity of speech, interruptions or hesitations (marked by "..."). There are no clear signs of interruptions or hesitations in the text.*

*- Note the use of present tense and vivid descriptions. The narrator uses vivid descriptions to recount their experience.*

*- Identify figures of speech, metaphors or euphemisms. No specific figures of speech, metaphors, or euphemisms were identified.*

*- Look for signs of dissociation and persistent negative emotions. There are no clear indications of dissociation or persistent negative emotions in the text.*

*List of relevant verbatims: []*

*6- Conclusion:*

*#diagnosis# Non-PTSD*

*The narrator's account shows that they were able to cope with their traumatic experience and find safety, although it was a harrowing ordeal. They did not exhibit clear symptoms of PTSD, but the narrative is still filled with vivid details and emotional intensity.”*

# Bibliography

[Elor, Yotam, et Hadar Averbuch-Elor. 2022. « To SMOTE, or not to SMOTE? » arXiv. https://doi.org/10.48550/arXiv.2201.08528.](https://www.zotero.org/google-docs/?3U85rZ)

[Jiang, Albert Q., Alexandre Sablayrolles, Arthur Mensch, Chris Bamford, Devendra Singh Chaplot, Diego de las Casas, Florian Bressand, et al. 2023. « Mistral 7B ». arXiv. https://doi.org/10.48550/arXiv.2310.06825.](https://www.zotero.org/google-docs/?3U85rZ)

[Lin, Tsung-Yi, Priya Goyal, Ross Girshick, Kaiming He, et Piotr Dollár. 2018. « Focal Loss for Dense Object Detection ». arXiv. http://arxiv.org/abs/1708.02002.](https://www.zotero.org/google-docs/?3U85rZ)

[Yang, Kailai, Shaoxiong Ji, Tianlin Zhang, Qianqian Xie, Ziyan Kuang, et Sophia Ananiadou. 2023. « Towards Interpretable Mental Health Analysis with Large Language Models ». https://doi.org/10.48550/ARXIV.2304.03347.](https://www.zotero.org/google-docs/?3U85rZ)
